# Laparoscopic treatment of ventral hernias: the Italian national guidelines

**DOI:** 10.1007/s13304-023-01534-3

**Published:** 2023-05-22

**Authors:** Fabio Cesare Campanile, Mauro Podda, Francesca Pecchini, Marco Inama, Sarah Molfino, Marco Augusto Bonino, Monica Ortenzi, Gianfranco Silecchia, Ferdinando Agresta, Michela Cinquini, the Italian Laparoscopic Ventral Hernia Guideline Group, Ferdinando Agresta, Roberto Cirocchi, Micaela Piccoli, Nereo Vettoretto, Fabio Cesare Campanile, Michela Cinquini, Elena Albanese, Luca Ansaloni, Armando Antinori, Paolo Baccari, Rossana Berta, Graziano Ceccarelli, Diego Cuccurullo, Francesco Diomede, Clelia Esposito, Giovanni Carlo Ferrari, Guendalina Graffigna, Roberta Monzani, Stefano Olmi, Tiziana Placidi, Marco Augusto Bonino, Marco Inama, Sarah Molfino, Francesca Pecchini, Mauro Podda, Alice Clerici, Victor Radu, Gianfranco Silecchia

**Affiliations:** 1 Division of General Surgery, ASL Viterbo, San Giovanni Decollato—Andosilla Hospital, Civita Castellana, Italy; 2 Department of Surgical Science, University of Cagliari, Cagliari, Italy; 3 Department of General Surgery, Emergency and New Technologies, Baggiovara General Hospital, AOU Modena, Modena, Italy; 4 General and Mininvasive Surgery Department, Pederzoli Hospital, Peschiera del Garda, Verona, Italy; 5 General Surgery Unit Chirurgia III, ASST Spedali Civili di Brescia, Brescia, Italy; 6 Department of Surgery, Geneva University Hospitals, Geneva, Switzerland; 7 Department of General and Emergency Surgery, Marche Polytechnic University, Via Conca 71, 60126 Ancona, Italy; 8 Department of Medical-Surgical Sciences and Translation Medicine, Faculty of Medicine and Psychology, Sapienza University of Rome, S. Andrea Hospital, Rome, Italy; 9 UOC Chirurgia Generale, Ospedale di Vittorio Veneto, Treviso, Italy; 10 Department of Oncology, Laboratory of Methodology of Sistematic Reviews and Guidelines Production, Istituto di Ricerche Farmacologiche Mario Negri IRCCS., Milan, Italy

**Keywords:** Italian Guidelines, Laparoscopic ventral hernia repair, Minimally invasive ventral hernia repair

## Abstract

Primary and incisional ventral hernias are significant public health issues for their prevalence, variability of professional practices, and high costs associated with the treatment In 2019, the Board of Directors of the Italian Society for Endoscopic Surgery (SICE) promoted the development of new guidelines on the laparoscopic treatment of ventral hernias, according to the new national regulation. In 2022, the guideline was accepted by the government agency, and it was published, in Italian, on the SNLG website. Here, we report the adopted methodology and the guideline’s recommendations, as established in its diffusion policy. This guideline is produced according to the methodology indicated by the SNGL and applying the Grading of Recommendations, Assessment, Development, and Evaluations (GRADE) methodology. Fifteen recommendations were produced as a result of 4 PICO questions. The level of recommendation was conditional for 12 of them and conditional to moderate for one. This guideline's strengths include relying on an extensive systematic review of the literature and applying a rigorous GRADE method. It also has several limitations. The literature on the topic is continuously and rapidly evolving; our results are based on findings that need constant re-appraisal. It is focused only on minimally invasive techniques and cannot consider broader issues (e.g., diagnostics, indication for surgery, pre-habilitation).

**Supplementary Information:**

The online version contains supplementary material available at 10.1007/s13304-023-01534-3.

## Background

Primary and incisional ventral hernias are significant public health issues for their prevalence, variability of professional practices, and high costs associated with the treatment. Abdominal wall surgery for primary and incisional ventral hernia repair can also imply long and painful periods of illness, absence from work, and possible adverse outcomes. Surgery can be highly challenging due to the size of the hernia defect, prolonged operative time, extent of adhesions, and risk of iatrogenic bowel injuries. The complexity of the evidence available in the literature and its fragmentation make it difficult for a surgeon to determine the best possible treatment options.

Different minimally invasive options (laparoscopic and now also robotic) have been introduced along with several open techniques in an extreme variability that concerns not only the access for the defect repair but also the physio-pathological assumptions underlying it. A multi-institutional study analyzed the variability in the techniques used in different hospitals and demonstrated that the differences in the outcomes (especially hernia recurrence) among the different institutions were related to the techniques adopted [1]. Furthermore, a recent survey showed considerable heterogeneity in the treatment choices of Danish experts in abdominal wall surgery confronted with 25 clinical cases [2]. Finally, a recent study has highlighted considerable variability in the attitude of the experts, with the impossibility of reaching a consensus in different areas [3].

The incidence of primary and incisional ventral hernias in Italy can be estimated at 17,000 cases per year. In 2019, there were 78,182 hospitalizations (acute, long-term care, and rehabilitation) with the primary diagnosis of ventral hernia; 68.9% were associated with a surgical operation, of which 79.5% were in elective and 20.5% in ambulatory settings. Currently, ventral hernias account for 64.9% of the series and umbilical for the remaining 35.1%. At least one-third of ventral hernias are operated on urgent/emergent settings, and 13.1% of the operations are performed laparoscopically. The average hospital stay for elective cases is 6.8 days, 5 for laparoscopic, and 7.1 days for open surgery [4].

In January 2010, the first Italian Consensus Conference on the subject was organized. In 2015, the Board of Directors of the Italian Society for Endoscopic Surgery (SICE) promoted the development of international guidelines on the laparoscopic treatment of primary and incisional ventral hernias under the auspices of the European Association for Endoscopic Surgery (EAES) and the European Hernia Society (EHS) [5].

Since 2017, Italian law established that, taking into account the case’s specificities, health professionals must comply with the recommendations provided by the guidelines collected in a national database (SNLG—Sistema Nazionale Linee Guida—Italian National Guideline System). Public and private bodies and institutions, and technical–scientific societies, registered in a special list established and regulated by a government agency (ISS—Istituto Superiore di Sanità), may elaborate guidelines to be published on the SNLG.

In 2019, the Board of Directors of the Italian Society for Endoscopic Surgery (SICE) promoted the development of new guidelines on the laparoscopic treatment of ventral hernias, according to the new national regulation. In 2022, the guideline was accepted by the government agency, and it was published, in Italian, on the SNLG website [6].

Here, we report the adopted methodology and the guideline’s recommendations, as established in its diffusion policy.

## Methods

### Objectives of the guidelines

The guideline recommendations are intended for all healthcare professionals involved in the treatment of adult patients with primary and incisional ventral hernias.

This guideline aims to be a tool to:

1. Improve and standardize the clinical practice concerning the laparoscopic treatment of primary and incisional ventral hernias in Italy, indicating to health practitioners involved in the care of ventral hernias the most effective and safe methods to repair them and reducing the variability of practices in our country.
2. Offer the patient the opportunity to take advantage of optimized and personalized therapeutic paths based on scientific evidence.
3. Offer a reference basis on the available evidence.

This guideline does not mean examining all aspects of ventral hernia treatment: it concentrates on the role of the minimally invasive techniques for ventral hernia in the broader scenario of abdominal wall surgery.

It has recently been hypothesized that the results of the treatment of incisional and primary ventral hernias should not be evaluated cumulatively since more treatment difficulties and worse outcomes would burden the former [7, 8]. This position is, however, still controversial [9, 10]. While being aware that this may be a confounding factor, in this guideline, we decided to assess the two conditions cumulatively, having noted that most of the available literature does not consider their outcomes separately.

This guideline is produced according to the methodology indicated by the SNGL. Applying the Grading of Recommendations, Assessment, Development, and Evaluations (GRADE) methodology [11], the recommendations resulting from the balance between favorable and unfavorable effects of therapeutic alternatives also consider the patient's values and preferences, available resources, equity, acceptability, and feasibility. In the presence of relative uncertainty regarding the superiority of one intervention over another, the panel makes a "conditional" (or weak) recommendation in favor or against a specific treatment. This recommendation should not be read as an obligation but instead leaves ample room for the decision-making process that should be shared between the surgeon and the patient for consideration of the patient's preferences and values, as well as individual circumstances and characteristics. Similarly, even "strong" recommendations cannot be interpreted as "gold standards of care" since they must take into account the unique circumstances and preferences of the individual patient, as well as the availability of equipment, surgical skills, and staff experience for each type of treatment.

The contents of this document were reported following the guidelines developed by the Appraisal of Guidelines for Research and Evaluation (AGREE) group using the AGREE Reporting tool [12].

The Board of Directors of SICE appointed a qualified expert panel with experience in the production and coordination of guidelines. It also made available the technical and organizational secretariat and the funds necessary for the project implementation. Finally, SICE involved the leading Italian surgical scientific societies (Italian Society of Surgery—SIC, Italian Association of Hospital Surgeons—ACOI, Italian Society of Hernia and Abdominal Wall Surgery—ISHAWS), each of which indicated a member of the Scientific Technical Committee (CTS). The CTS contributed to the definition of the scope of the guideline, defined the policy of assessment and management of conflicts of interest, and defined the explicit criteria for the selection of the panel members (relevant clinical practice in abdominal wall surgery, scientific production published in national and international journals with high impact on the topics covered by the guideline, previous participation in the development of guidelines); and the evidence review team (ERT) (experience in bibliographic research and assessment of scientific literature); identified the guideline production group, the chair and the methodological co-chair of the guideline. The multidisciplinary and multi-professional experts' panel included general and abdominal wall surgeons, anesthesiologists, and nurses. In addition, it was integrated with two patient representatives and a patient advocate, who participated in the entire production process, including the definition of the final recommendations.

### Guidelines development

The guideline kick-off meeting took place in Rome (Italy) on July 19, 2019. All participants’ relevant or potentially relevant conflicts of interest were made public and discussed during the meeting. The objectives of the guideline, the tasks, and the expected timetable were presented. An intermediate meeting took place in Ancona (Italy) on September 29th, 2019. The rest of the process, during the SARS-CoV-2 pandemic, was completed by online tools. The management of declared conflicts of interest concerning all those involved in any capacity in developing the guideline was based on the principles outlined by the Guidelines International Network [13] and detailed in the methodological manual for the production of clinical practice guidelines by the National Center for Clinical Excellence, Quality and Safety of Care (CNEC) [14].

### PICO question development

The panel formulated the questions addressed in this guideline following the PICO criteria (Population, Intervention, Comparison, and Outcome). Several outcomes of interest for each question were identified, and their relative relevance was graded as follows [11, 13]:

- Essential outcomes (also referred to as "critical");
- Important but not essential outcomes;
- Irrelevant outcomes.

Only "critical" or "important" outcomes were considered in the literature review and subsequently in the formulation of recommendations, according to the criteria shown in Table 1.

**Table 1:** Relevance of the Outcome measures according to GRADE (Grading of Recommendations, Assessment, Development, and Evaluations)

Rating (Median)	Relevance	Included in
*7–9*	Critical outcome	Included into the evidence table/summary of findings table Included into the Recommendations
*4–6*	Important but not critical	Included into the evidence table/summary of findings table Not included into the Recommendations
*1–3*	Of limited importance	Not included into the evidence table/summary of findings table Not Included into the Recommendations

From: https://gdt.gradepro.org/app/handbook

### Literature search

**Fig. 1:**
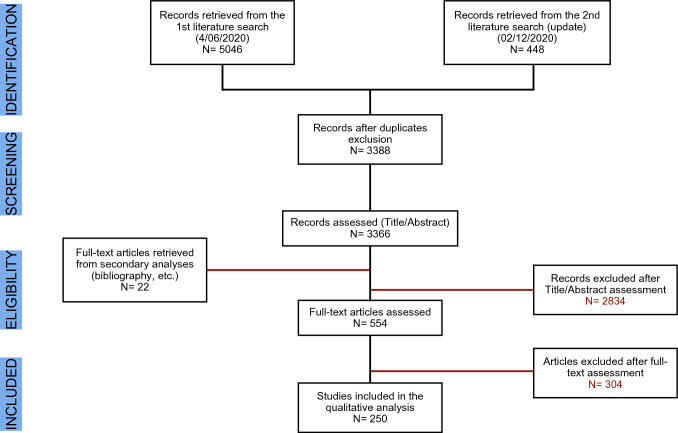
PRISMA Flow-diagram

The first literature search was conducted to identify existing guidelines on the subject. The quality of the guidelines retrieved with the search was assessed, and two were found acceptable [5, 15]. Both guidelines, however, needed reference updates. A systematic literature search was therefore performed for studies published from January 1, 2000, to June 4, 2020, aimed at identifying the systematic reviews (with or without meta-analysis) and the primary studies (randomized and non-randomized trials) relating to the efficacy and safety of treatments, costs, effectiveness, and those relating to patient values and preferences (Fig. 1). Search strategies are reported as supplementary material (Appendix 1). The search was conducted on PubMed, Embase, Cochrane Library, and Web of Science. On December 2, 2021, the same search strings were used to update the literature published up to June 5, 2020.

The list of titles and abstracts obtained from querying the databases was screened on Rayyan© to select the relevant articles to each question. Then, two members of the ERT examined each element separately, determining its inclusion or exclusion based on pre-established criteria. In case of disagreement between the two team members, the inclusion or exclusion of the article was determined after mutual discussion and querying the methodological chair.

Case reports, trial protocols, narrative reviews and summaries, letters, editorials, position papers, congress abstracts, and posters were excluded. The selected articles were examined in full text and further analyzed to determine their inclusion. In the absence of systematic reviews of randomized and non-randomized controlled studies, or in the presence of systematic reviews judged to be of low methodological quality according to the AMSTAR II tool [16], primary studies were analyzed, and new meta-analyses, wherever possible, were performed by the ERT to assess specific outcomes. The methodological quality of the selected guidelines judged to be reliable was assessed with AGREE II [12]. The methodological quality of the systematic reviews was assessed using the AMSTAR II checklist [16]. The risk of bias in randomized trials was assessed using the Cochrane criteria [17] and that of observational studies with the Newcastle–Ottawa Scale [18].

For each outcome considered in the clinical questions, the working group assessed the confidence in effect estimates based on five dimensions (risk of bias, inconsistency, indirectness, imprecision of the estimated effects, and publication bias) [19, 20]. The confidence in effect estimates was summarized in four levels (high, moderate, low, and very low).

### Synthesis of the evidence and development of the recommendations

The working group summarized the efficacy and safety of the interventions in synoptic tables reporting the study's general characteristics and the summary of the effects with an indication of their extent and quality.

The panel applied the GRADE methodology, which provides the overall assessment of the relationship between desirable and undesirable effects through the "Evidence to Decision Framework" [21, 22].

For strong recommendations, the panel concluded that most of the patients who receive the intervention covered by the recommendation obtain a benefit from it, and the benefits outweighed the risks. Conversely, for conditional recommendations, the positive effects probably prevail over the harmful ones (or vice versa for negative recommendations), but there is still significant uncertainty in this regard. For the reporting of the recommendations, it was agreed to use standard expressions such as: "the panel recommended… " (strong recommendation), "the panel suggested …" (conditional recommendation), "the panel suggested that one strategy be used or not" (conditional recommendation neither for nor against).

The final recommendations were proposed in a preliminary version by the Working Group and were then discussed by the panel. Finally, the panel formally voted on the final versions of the recommendations through an online tool. All the recommendations in this guideline were "conditional" since the literature on the subject is characterized by low confidence in effect estimates, without any condition that warrants a "strong" recommendation in the presence of low or very low-quality evidence.

### Economic analysis

The process of selecting the literature and its evaluation included studies determining the economic impact of the interventions examined by the guideline. The Working Group considered studies reporting complete economic analyses, such as cost–benefit, cost-utility, or cost-effectiveness analyses. Conversely, simple cost analyses were not considered [23].

### External review

After approval by the panel, a draft version of the guideline was sent to external experts for a revision of the contents and methods. The reviewers were chosen from a pool of international experts of a well-known authority on the topics covered by the guideline, with relevant clinical practice, scientific production published in national and international journals with a high impact on the topics covered, and previous experience in guidelines methodology.

### Update, diffusion, and implementation of the guidelines

Due to the continuous and rapid evolution of scientific knowledge and technical innovations on the subject of the guideline, its update is expected within two years (January 2024). Future updates will be performed through a systematic review of the literature to verify the release of new evidence that may influence the strength and direction of the recommendations.

The monitoring of the application of the present guideline can be carried out considering a benchmark of ≥ 50%. Any findings of indicators below the suggested benchmark may represent grounds for a review of the recommendation or analysis of the non-implementation.

## Results

The summary of the Guideline recommendations is reported in Table 2.

**Table 2:** Summary of the 2022 Italian guidelines on the laparoscopic treatment of primary and incisional ventral hernias

	PICO Question	Recommendation	Strength of recommendation
1A	In patients with a primary or incisional ventral hernia (P), is it preferable to choose laparoscopic (I) or open (C) surgery in terms of (O) mortality, morbidity, recurrence, quality of life, length of hospitalization, postoperative pain, and costs?	For treating patients with a primary or incisional ventral hernia in the general population, the panel suggested that laparoscopic surgery be used as an alternative to open surgery for hernia defects smaller than 10 cm	Conditional, based on LOW confidence in effect estimates for mortality, and MODERATE confidence in effect estimates on the other outcomes
1B	In elderly patients with primary or incisional ventral hernia (P), is it preferable to choose laparoscopic (I) or open (C) surgery in terms of (O) mortality, morbidity, recurrence, quality of life, length of hospitalization, postoperative pain, and costs?	For the treatment of elderly patients (> 70 years) who require surgery for primary or incisional ventral hernia, the panel suggested that laparoscopic treatment be used as an alternative to open surgery	Conditional, based on VERY LOW confidence in effect estimates
1C	In people with obesity (body mass index ≥ 30 kg/m^2^) and primary or incisional ventral hernia (P) is it preferable to choose laparoscopic (I) or open (C) surgery in terms of (O) mortality, morbidity, recurrence, quality of life, length of hospitalization, postoperative pain, and costs?	The panel suggested that laparoscopic treatment be used as an alternative to open surgery for primary or incisional ventral hernias in patients with obesity	Conditional, based on LOW confidence in effect estimates
1D	In the emergency treatment of patients with a primary or incisional ventral hernia (P) is it preferable to choose laparoscopic (I) or open (C) surgery in terms of (O) mortality, morbidity, and recurrence?	The panel suggested that laparoscopic surgery be used as an alternative to open surgery for the treatment of patients with a primary or incisional ventral hernia in emergency settings	Conditional, based on VERY LOW confidence in effect estimates for mortality, morbidity and recurrence
1E	In treating patients with a border primary or incisional hernias (P) is it preferable to choose laparoscopic (I) or open (C) surgery in terms of (O) mortality, morbidity, recurrence, quality of life, length of hospitalization, postoperative pain, and costs?	The panel cannot provide a recommendation about the treatment of patients with a border primary or incisional hernia, due to insufficient confidence in the effect estimates	No recommendation. The confidence in effect estimates was VERY LOW
1F	In patients with a parastomal hernia (P) is it preferable to choose laparoscopic (I) or open (C) surgery in terms of (O) mortality, morbidity, recurrence, quality of life, length of hospitalization, postoperative pain, and costs?	The panel suggested that laparoscopic surgery be used as an alternative to open treatment for patients with a parastomal hernia	Conditional, based on VERY LOW confidence in effect estimates
2A	In the laparoscopic treatment of patients with primary or incisional ventral hernia (P) is it preferable to close the defect (IPOM plus) (I) or not (IPOM) (C) in terms of (O) mortality, morbidity, recurrence, quality of life, length of stay, postoperative pain, and costs?	The panel suggested that the hernia defect be sutured in the laparoscopic treatment of patients with primary or incisional ventral hernia	Conditional, based on VERY LOW confidence in effect estimates
2B	In patients undergoing minimally invasive surgery for ventral or incisional hernia (P), is a robotic (I) or laparoscopic (C) approach preferable in terms of (O) mortality, morbidity, recurrence, quality of life, length of stay, postoperative pain, costs?	For patients with primary or incisional ventral hernia, the panel suggested that either laparoscopic or robotic techniques be used	Conditional, based on LOW confidence in effect estimates
2C	In patients undergoing laparoscopic parastomal hernia repair (P), is the Keyhole (I) or Sugarbaker (C) technique preferable in terms of (O) mortality, morbidity, recurrence, quality of life, length of stay, postoperative pain, and costs?	For the laparoscopic treatment of parastomal hernias, the panel suggested that the Sugarbaker technique be used rather than the keyhole technique	Conditional, based on VERY LOW confidence in effect estimates
2D	For the laparoscopic repair of patients with ventral or incisional hernia (P), is it preferable to fix the mesh with sutures (I) or mechanical tacks (C) in terms of (O) mortality, morbidity, recurrence, quality of life, length of stay, postoperative pain, and costs?	For the laparoscopic treatment of patients with primary or incisional ventral hernia, the panel suggested that the mesh be fixed by mechanical tacks rather than transfixed sutures	Conditional, based on VERY LOW confidence in effect estimates
2E	For the laparoscopic treatment of patients with ventral or incisional hernia (P), is it preferable to fix the mesh with absorbable (I) or non-absorbable (C) fixation devices in terms of (O) mortality, morbidity, recurrence, quality of life, length of stay, postoperative pain, and costs?	For the laparoscopic treatment of patients with primary or incisional ventral hernia, the panel suggested that the mesh be fixed either with absorbable or permanent devices	Conditional, based on VERY LOW confidence in effect estimates
2F-A	For the laparoscopic treatment of patients with ventral or incisional hernia (P) with the IPOM technique, what is the optimal overlap of the mesh on the abdominal wall surface (I) (C) in terms of (O) mortality, morbidity, recurrence, quality of life, length of stay, postoperative pain, and costs?	In the treatment of patients with primary or incisional ventral hernia, with a defect diameter of 4 cm or larger, the panel suggested a minimum overlap of the mesh beyond the margins of the defect of 5 cm on each side	Conditional, based on VERY LOW confidence in effect estimates
2F-B	For the laparoscopic treatment of patients with ventral or incisional hernia (P) with the IPOM technique, what is the optimal overlap of the mesh on the abdominal wall surface (I) (C) in terms of (O) mortality, morbidity, recurrence, quality of life, length of stay, postoperative pain, and costs?	In the treatment of patients with primary or incisional ventral hernia, with a defect less than 4 cm, the panel suggested a minimum overlap of the mesh beyond the margins of the defect of 3–5 cm on each side	Conditional based on VERY LOW confidence in effect estimates
3A	In patients operated with a minimally invasive approach for primary or incisional ventral hernia, or parastomal hernia (P), is deep neuromuscular block (I) preferable to a moderate one (C) (TOF > 0), in terms of (O) success of the procedure, evaluation of the intra-abdominal workspace, postoperative pain, operator satisfaction, and difficulty of the procedure?	The panel could not issue any recommendation in favor or against deep neuromuscular block	No recommendation. The confidence in effect estimates in this research field was VERY LOW
4A	In patients operated on for primary or incisional ventral or parastomal hernia with a minimally invasive approach (P), is it preferable to combine (I) or not to combine (C) an analgesic loco-regional anesthesia with general anesthesia in terms of (O) success of the procedure, evaluation of the intra-abdominal workspace, postoperative pain, operator satisfaction, and difficulty of the procedure?	In the laparoscopic treatment of patients with a primary or incisional ventral hernia, the panel suggested that regional anesthesia be associated with general anesthesia	Conditional to MODERATE confidence in effect estimates

### RESEARCH QUESTION 1: In terms of efficacy and safety, is laparoscopic surgery preferable to open surgery to treat patients with a primary or incisional ventral hernia?

The panel compared laparoscopic and open techniques in the general population of patients with a primary or incisional ventral hernia and also in different subgroups whose specific general or local conditions could potentially change the risk–benefit ratio for the two techniques (elderly, people with obesity). It also considered the indications in specific anatomical or pathophysiological conditions that could impact the treatment choice or its outcome (interventions performed in emergency settings, parastomal or border hernias).

#### Laparoscopic vs. open surgery

**PICO 1A.** In patients with a primary or incisional ventral hernia (P), is it preferable to choose laparoscopic (I) or open (C) surgery in terms of (O) mortality, morbidity, recurrence, quality of life, length of hospitalization, postoperative pain, and costs?

**RECOMMENDATION 1A.** For treating patients with a primary or incisional ventral hernia in the general population, the panel suggested that laparoscopic surgery be used as an alternative to open surgery for hernia defects smaller than 10 cm.

**STRENGTH OF RECOMMENDATION.** Conditional, based on LOW confidence in effect estimates for mortality, because deriving from a single observational study; and MODERATE confidence in effect estimates on the other outcomes, because deriving from meta-analyses of randomized controlled studies, many of which presented a high risk of bias.

**Evidence synthesis.** Our meta-analyses demonstrated a trend in favor of the laparoscopic approach for mortality, overall morbidity, and length of hospitalization, with low-quality evidence. For the other outcomes, we did not demonstrate an advantage for either technique. The panel evaluated the balance between desirable and undesirable effects in favor of the laparoscopic approach. The recommendation favoring the laparoscopic approach was conditioned not only by the low confidence in effect estimates but also by the size of the defect to be sutured (≤ 10 cm). Five systematic reviews and meta-analyses of randomized controlled trials comparing the laparoscopic and open techniques in patients with incisional and primary ventral hernias were identified [24–28]. The inclusion criteria varied between the reviews; in all of them, the maximum diameter included was 15 cm, but in most included cases it was ≤ 10 cm. The study by Sauerland et al*.* showed the highest methodological quality after evaluation with the AMSTAR II tool. It included ten randomized controlled trials [29–38]. Our updated analysis included two trials not available at the time of the publication by Sauerland et al*.* [39, 40]. We also performed two sensitivity analyses: excluding the trials at high risk of bias and considering only those with incisional hernias alone. We also examined our meta-analysis results against a large observational study of the Vizient database that compared 39,505 patients treated with an open technique with 6,829 treated with laparoscopy [41]. The randomized trials considered in the meta-analysis included almost exclusively patients with hernia defects ≤ 10 cm in diameter. For this reason, the panel limited the recommendation to hernia defects ≤ 10 cm in diameter. No systematic review performed a meta-analysis on mortality since the outcome was not present in the primary studies. The Vizient study showed a reduction in mortality in favor of the laparoscopic group (0.16% *vs.* 0.99%; RR 0.16, 95%CI 0.089–0.294) [***Confidence in effect estimates: low due to the observational design of the study***].

**Fig. 2:**
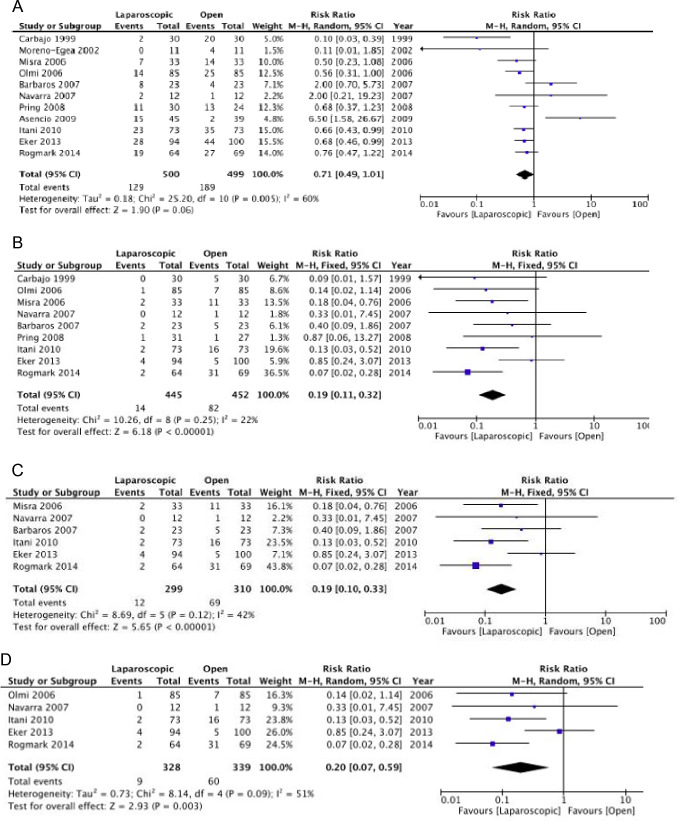
Laparoscopic vs. Open repair: Postoperative morbidity (**A**), Surgical-Site Infection—general (**B**), Surgical-Site Infection—low risk of bias (**C**), Surgical-Site Infection—incisional hernias only (**D**)

We showed a trend in favor of the laparoscopic approach for postoperative morbidity, with a 29% reduction in overall morbidity (25.8% *vs.* 37.9%; RR = 0.71, 95% CI 0.49–1.01;* I*^*2*^ = 60%) [***Confidence in effect estimates: low due to risk of bias and heterogeneity***] (Fig. 2). A meta-analysis of observational studies [42] and the Vizient study [41] also showed lower overall morbidity rates in the laparoscopic groups (OR 0.66, 95%CI 0.48–0.90; 3.5% *vs.* 11.4%; RR 0.31, 95%CI 0.273–0.352) [***Confidence in effect estimates: low due to observational design of the studies***]. Our meta-analysis showed an 82.9% reduction in the incidence of surgical site infections in favor of the laparoscopic approach (3.1% *vs.* 18.1%; RR 0.19, 95%CI 0.11–0.32; *I*^*2*^ = 22%). The sensitivity analyses performed after excluding the trials at high risk of bias and considering only those that excluded primary hernia confirmed the result (Fig. 2). The Vizient study also demonstrated a lower incidence of infections in patients operated on with laparoscopy (0.67% *vs.* 2.83%; RR 0.238, 95%CI 0.177–0.319) [***Confidence in effect estimates: high***].

**Fig. 3:**
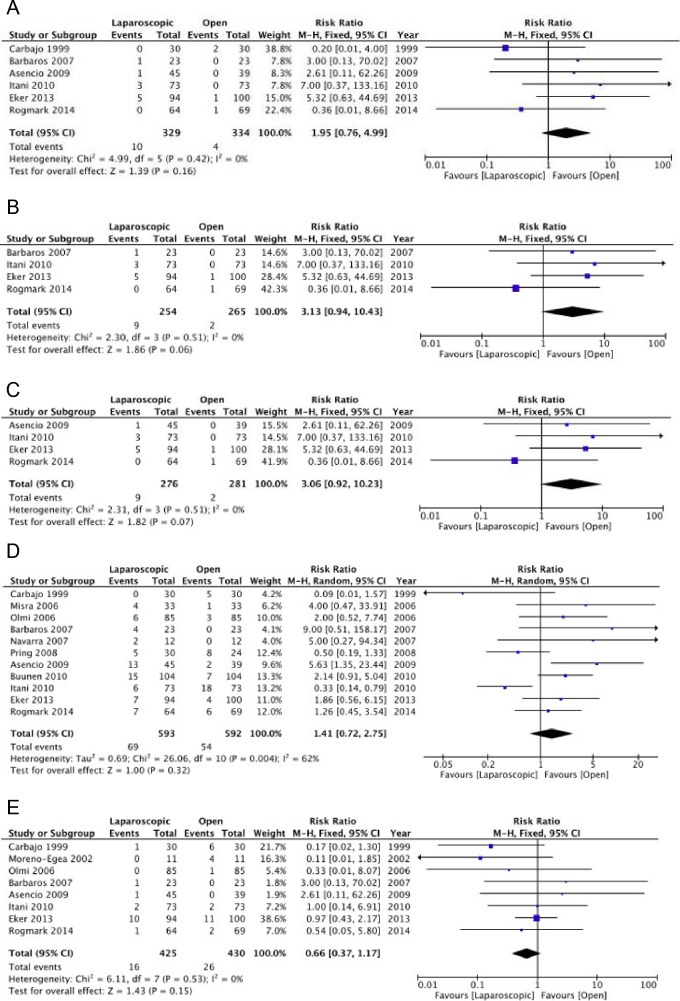
Laparoscopic vs. Open repair: Full-thickness enterotomies—general (**A**), Full-thickness enterotomies—low risk of bias (**B**), Full-thickness enterotomies—incisional hernias only (**C**), Seromas (**D**), Hematomas (**E**)

Our meta-analysis showed that the laparoscopic technique was associated with increased accidental full-thickness enterotomies. Similar results were also obtained in the two sensitivity analyses [***Confidence in effect estimates: low due to risk of bias and imprecision***] (Fig. 3).

**Fig. 4:**
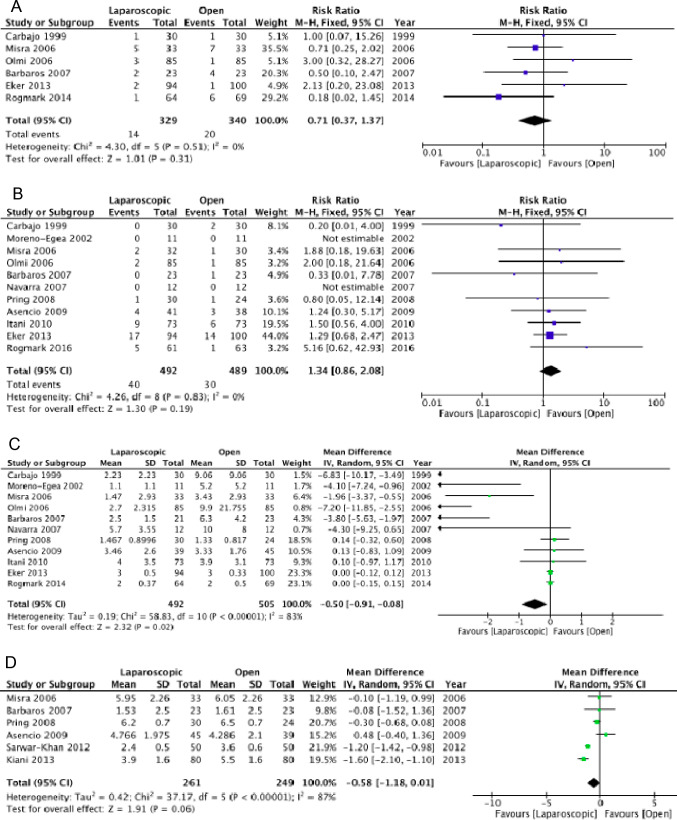
Laparoscopic vs. Open repair: Re-operations (**A**), Hernia recurrence (**B**), Length of hospital stay (**C**), Post-operative pain (**D**)

The incidence of seromas and hematomas did not differ between the two treatment groups (seromas RR 1.41, 95%CI 0.72–2.75; *I*^*2*^ = 62%; hematomas RR 0.66, 95%CI 0.37–1.17; *I*^*2*^ = 0%) [***Confidence in effect estimates: low due to risk of bias and heterogeneity***] (Fig. 3). The risk of re-operations did not differ between the two groups (RR 0.71, 95%CI 0.37–1.17; *I*^*2*^ = 0%) [***Confidence in effect estimates: low due to risk of bias and imprecision***] (Fig. 4). We did not find any difference in the recurrence rate between the laparoscopic and open techniques (RR 1.22, 95%CI 0.62–2.38; *I*^*2*^ = 0%) [***Confidence in effect estimates: moderate due to risk of bias***] (Fig. 4). Three randomized trials assessed the quality of life without significant differences between the two groups [34, 36, 37] [***Confidence in effect estimates: moderate due to risk of bias***]. The laparoscopic approach was associated with a shorter length of hospital stay [***Confidence in effect estimates: low due to risk of bias and heterogeneity***] (Fig. 4).

Our meta-analysis showed a decrease in postoperative pain in favor of the laparoscopic technique (MD − 0.58, 95%CI − 1.18 to 0.01; *I*^*2*^ = 87%) [***Confidence in effect estimates: low due to risk of bias and heterogeneity***] (Fig. 4).

The only complete economic analysis was published by Wolf et al*.* in 2018 [43]; it compared costs and outcomes in terms of QALYs for three alternatives of treatment: laparoscopic, open, and watchful waiting. This analysis demonstrated an advantage of laparoscopic surgery over open surgery.

**Patient values and preferences** No evidence was found in the literature. In the outcome prioritization phase, the patient representatives and patient advocate expressed their preferences in line with those of the rest of the panel. For this reason, the panel believed there was no variability or uncertainty about patients' values and preferences.

**Balance between desirable/undesirable effects.** Laparoscopic surgery proved beneficial in postoperative infection reduction, but it might have been disadvantageous in intraoperative bowel injury. The latter outcome, however, was assessed on an extremely small sample size (consisting of a few units). For mortality, overall morbidity, and length of hospitalization, the meta-analysis demonstrated only a trend in favor of the laparoscopic approach, with low evidence quality (observational studies) confirming its advantage. For the other outcomes considered, it was impossible to demonstrate an advantage for one or the other technique. Therefore, the panel evaluated the balance between desirable and undesirable effects in favor of the laparoscopic approach to reduce postoperative infections.

**Equity, acceptability, feasibility.** In the Italian health system context, the suggested approach has no equity and feasibility issues. However, regarding the acceptability by all stakeholders, there were concerns regarding a part of the surgical community that, based on their own experience, did not consider laparoscopic surgery acceptable or limited its implementation to more restricted indications.

**Note to the recommendation.** The panel made a conditional recommendation in favor of the laparoscopic approach due to the very low certainty of the evidence and the size of the defect to be repaired. Therefore, the preoperative path must include the evaluation of the defect with imaging methods (ultrasound, CT scan). The choice of intervention must also consider individual patient factors.

#### Elderly population

**PICO 1B.** In elderly patients with a primary or incisional ventral hernia (P), is it preferable to choose laparoscopic (I) or open (C) surgery in terms of (O) mortality, morbidity, recurrence, quality of life, length of hospitalization, postoperative pain, and costs?

**RECOMMENDATION 1B.** For the treatment of elderly patients (> 70 years) who require surgery for primary or incisional ventral hernia, the panel suggested that laparoscopic treatment be used as an alternative to open surgery.

**STRENGTH OF RECOMMENDATION.** Conditional based on VERY LOW confidence in effect estimates, deriving from observational studies burdened by prognostic imbalance and imprecision.

**Evidence synthesis.** There are no systematic reviews or randomized controlled trials assessing the role of laparoscopic ventral hernia repair in the elderly Two observational studies comparing the results of laparoscopic and open surgery in elderly patients (> 80 years, the first and > 70 years, the second) were analyzed. Both studies demonstrated an advantage for the laparoscopic approach in terms of reduction of the surgical site and systemic complications and length of hospital stay. Mortality was found to be lower in patients undergoing laparoscopic surgery in one of them (0.85% *vs.* 1.80; RR 0.47, 95%CI 0.19–1.16), whereas the other showed no statistical difference between the two groups (1.21% *vs.* 1.11%; RR 1.06, 95%CI 0.10–11.59) [***Confidence in effect estimates: very low due to risk of bias and imprecision***] [44, 45].

The first study demonstrated an advantage for laparoscopic surgery regarding overall morbidity (9.04% *vs.* 11.7%; RR 0.77, 95%CI 0.59–1.01), the incidence of surgical site infections (1.02% *vs.* 3.39%; RR 0.30, 95%CI 0.134–0.679) and pulmonary complications (2.21% *vs.* 4.41%; RR 0.50, 95%CI: 0.289–0.876) [44].

The second showed lower surgical site morbidity (15.47 *vs.* 22.90; RR 0.68, 95%CI 0.38–1.19), incidence of surgical site infections (1.19% *vs.* 11.73%; RR 0.10, 95%CI 0.01–0.74), systemic complications (15.47% *vs.* 33.51%; RR 0.46, 95%CI: 0.27–0.79) and intensive care unit admission (4.76% *vs.* 20.67%, RR 0.23, 95%CI 0.085–0.625). Symptomatic seromas (13.09% *vs.* 2.23%; RR 5.86, 95%CI 1.92–17.86) were more frequent in the laparoscopic group [***Confidence in effect estimates: very low due to the risk of bias and imprecision***]. In both studies, the length of hospitalization was shorter for patients in the laparoscopic group (3 ± 3.8 *vs.* 3.4 ± 6.2 days and 7.7 range 0–29 *vs.* 3.4 range 0–11 days) [***Confidence in effect estimates: very low due to the risk of bias and imprecision***]. The study by Spaniolas et al*.* did not investigate hernia recurrence, whereas, in the study by Neupane et al*.*, fewer recurrences were found in the laparoscopic group (7.14% *vs.* 9.49%, RR 0.75, 95%CI 0.31–1.84) [***Confidence in effect estimates: very low due to the risk of bias and imprecision***].

**Patient values and preferences.** No evidence was found in the literature. In the outcome prioritization phase, the patient representatives and patient advocate expressed their preferences in line with those of the rest of the panel. For this reason, the panel believed there was no variability or uncertainty about patients' values and preferences.

**Balance between desirable/undesirable effects.** In an elderly patient (> 70 years), laparoscopic surgery proved to be advantageous in terms of overall morbidity, both local (surgical site infections) and systemic. The incidence of seromas was the only morbidity parameter disadvantageous for the laparoscopic technique in one observational study with a low sample size. The panel evaluated the balance between desirable and undesirable effects in favor of the laparoscopic approach.

**Equity, acceptability, and feasibility.** The suggested approach has no equity, acceptability, or feasibility issues in the Italian health system context.

**Note to the recommendation.** The panel issued a recommendation in favor of the laparoscopic approach conditioned not only by the very low certainty of the effects but also by the high variability in general conditions and operative risk in the elderly population. Therefore, the choice of intervention in each case must consider the patient's individual factors and local characteristics. The indication of the 70-year threshold for the recommendation was motivated by the demographic characteristics of the patients included in the published studies that supported the recommendation and cannot be regarded as a definite limit. The diameter of the defect already expressed for question 1A must also be considered in this subgroup of patients.

#### Patients with obesity

**PICO 1C.** In people with obesity (body mass index ≥ 30 kg/m^2^) and primary or incisional ventral hernia (P), is it preferable to choose laparoscopic (I) or open (C) surgery in terms of (O) mortality, morbidity, recurrence, quality of life, length of hospitalization, postoperative pain, and costs?

**RECOMMENDATION 1C.** The panel suggested that laparoscopic treatment be used as an alternative to open surgery for primary or incisional ventral hernias in patients with obesity.

**STRENGTH OF RECOMMENDATION.** Conditional, based on LOW confidence in effect estimates, deriving from observational studies.

**Evidence synthesis.** The literature search did not retrieve systematic reviews or randomized trials. However, several registry studies with large study groups compared the outcomes of open *vs.* laparoscopic repair for people with obesity and a primary or incisional ventral hernia. Between 2017 and 2019, Owei et al*.* [46, 47] assessed the influence of BMI on patients undergoing surgery for primary or incisional ventral hernia registered on the ACS-NSQIP database between 2005 and 2015. The authors extracted the results of the laparoscopic and open technique from the same database (with identical inclusion and exclusion criteria). The studies included 102,191 patients operated on with an open technique (of which 59,806 with a BMI ≥ 30) and 55,180 operated on by laparoscopy (of which 35,551 with a BM I ≥ 30). Moreover, Fekkes et al*.* [48] and Regner et al*.* [49] published two studies from the same database, limited to 2011 the first and 2009–2012 the latter. The studies by Lee et al*.* [50], Froelich et al*.* [51], and Alizai et al*.* [52] were also considered.

From the analysis by Owei et al*.* [46, 47], patients with obesity undergoing laparoscopic surgery had lower morbidity rates compared to those operated with an open technique (4.2% *vs.* 12.61%, RR 0.28; 95%CI: 0.27–0.30). Surgical-site morbidity was also lower (superficial and deep surgical site infections, wound disruption): 1.96% *vs.* 8.54%, RR 0.22, 95%CI 0.20–0.23. A difference was also found in all components of local morbidity: abdominal wall infections (0.79% *vs.* 4.05%, RR 0.19, 95%CI 0.16–0.21), deep infections, organ infections, and abdominal wall dehiscences. Systemic morbidity was also lower for the laparoscopic technique (overall morbidity: 2.87% *vs.* 6.14%, RR 0.61, 95%CI 0.57–0.65; pulmonary embolism, acute renal failure, and urinary tract infection). On the other hand, no difference emerged for heart attacks and strokes [46, 47]. A higher morbidity rate (6.3% *vs.* 13.7%) for open surgery was also found in the studies by Lee et al*.* [50] (13.69% *vs.* 6.29%, RR 0.46, 95%CI 0.42–0.50), Froelich et al*.* [51], and Alizai et al*.* [52] [***Confidence in effect estimates: low due to the observational design of the studies***].

The only study to compare the recurrence rate in laparoscopic *vs.* open surgery in patients with obesity was that by Froylich et al*.*, [35] which, on an average follow-up of 50.7 months in the first group and 62.3 in the second, showed an incidence of 20.0 *vs.* 27.1% (RR = 0.74, 95%CI 0.36–1.50) [***Confidence in effect estimates: low due to the observational design of the studies***]. Fekkes et al*.* [48] showed that laparoscopic surgery had a shorter hospital stay (2.72 ± 7.93 *vs.* 1.86 ± 4.70 days).

**Patient values and preferences.** No evidence was found in the literature. In the outcome prioritization phase, the patient representatives and patient advocate expressed their preferences in line with those of the rest of the panel. For this reason, the panel believed there was no variability or uncertainty about patients' values and preferences.

**Balance between desirable/undesirable effects.** Laparoscopic surgery in a patient with obesity proved beneficial for all outcomes examined. The literature analyzed did not highlight any undesirable effects.

**Equity, acceptability, and feasibility.** The suggested approach has no equity, acceptability, or feasibility issues in the Italian health system context.

**Note to the recommendation.** The panel made a recommendation in favor of the laparoscopic approach in the patient with obesity, conditioned by the low certainty of the effects and the variability of general and local conditions in this population group. In patients with obesity the laparoscopic approach is privileged but the choice of intervention in each case must consider the patient's individual factors and local characteristics. The considerations on the diameter of the defect already expressed for question 1A must also be kept in mind in this segment of the population.

#### Emergency conditions

**PICO 1D.** In the emergency treatment of patients with a primary or incisional ventral hernia (P), is it preferable to choose laparoscopic (I) or open (C) surgery in terms of (O) mortality, morbidity, and recurrence?

**RECOMMENDATION 1D.** The panel suggested that laparoscopic surgery be used as an alternative to open surgery for treating patients with a primary or incisional ventral hernia in emergency settings.

**STRENGTH OF RECOMMENDATION.** Conditional, based on VERY LOW confidence in effect estimates.for mortality, morbidity, and recurrence, deriving from a comparative observational study based on registry data (risk of selection bias), whose groups were balanced with a matching method.

**Evidence synthesis.** An observational study on patients treated for primary or incisional ventral hernia in an emergency setting (with bowel obstruction or gangrene) [53] included 1,642 patients divided into two groups (laparoscopic and open), balanced with the propensity score-matched method based on demographic characteristics (age, sex, BMI, ASA, presence of preoperative sepsis, classification of wounds and comorbidities). The mortality rate was 1.3% in the laparoscopic group and 1.1% in the open group (RR 1.22, 95%CI 0.51–2.93). In the univariable analysis, the laparoscopic approach showed an overall morbidity rate of 9.1% *vs.* 15.1% of the open approach (RR 0.60, 95%CI 0.46–0.79). Abdominal wall complications (superficial and deep wound infections, dehiscence) were 3.0% *vs.* 7.9% (RR 0.38, 95%CI 0.24–0.60). There was no difference in the incidence of complications not associated with the surgical site (7.1% *vs.* 9.3%, RR 0.76, 95%CI 0.55–1.06). The study also highlighted that the incidence of unrecognized accidental enterotomies, albeit low, was higher in the laparoscopic group (0.7% *vs.* 0.0%). However, no bowel resection was performed at the 30-day follow-up after surgery. The multivariable analysis confirmed that the laparoscopic approach was independently associated with a lower incidence of abdominal wall complications (OR 0.35, 95%CI 0.22–0.57). The length of hospitalization in the laparoscopic group was 3.65 ± 5.88 *vs.* 4.33 ± 5.21 days in the open group [***Confidence in effect estimates: very low due to the observational design of the studies***].

**Patient values and preferences.** No evidence was found in the literature. In the outcome prioritization phase, the patient representatives and patient advocate expressed their preferences in line with those of the rest of the panel. For this reason, the panel believed there was no variability or uncertainty about patients' values and preferences.

**Balance between desirable/undesirable effects.** In an emergency setting, the overall lower morbidity and most of its components were desirable effects of laparoscopy. However, a higher incidence of accidental enterotomies was found after laparoscopic surgery, although no bowel resections were reported within 30 days following it. Therefore, the panel evaluated the balance between desirable and undesirable effects in favor of the laparoscopic approach.

**Equity, acceptability, and feasibility.** The suggested approach has no equity, acceptability, or feasibility issues in the Italian health system context.

**Note to the recommendation.** The panel formulated a recommendation in favor of the emergency laparoscopic approach conditioned not only by the very low certainty of the effects but also by the variability of the local and general conditions in this population group. Therefore, the choice of intervention in each case must consider the patient's individual factors, the general condition and the team's proficiency with emergency laparoscopy. The diameter of the defect already expressed for question 1A must also be considered in this subgroup of patients.

#### Border hernias

**PICO 1E.** In treating patients with a border primary or incisional hernias (P), is it preferable to choose laparoscopic (I) or open (C) surgery in terms of (O) mortality, morbidity, recurrence, quality of life, length of hospitalization, postoperative pain, and costs?

**RECOMMENDATION 1E.** The panel cannot issue a recommendation about the treatment of patients with a border primary or incisional hernia due to insufficient confidence in the effect estimates.

**STRENGTH OF RECOMMENDATION.** No recommendation. The confidence in effect estimates was VERY LOW, as it derived from a single observational study with a small sample size (risk of imprecision) and a high risk of bias. It was impossible to balance desirable and undesirable effects, and the confidence in the estimates was too low to release a recommendation.

**Evidence synthesis.** The literature that considered the laparoscopic treatment of border incisional hernias (epigastric, lumbar, suprapubic, and subcostal) included only case series that investigated the feasibility of laparoscopic surgery and compared its results with external controls of similar cases treated with an open technique. The only comparative, single-center, observational study included 55 patients with lumbar hernia operated on between 1995 and 2008 [54]. This study showed a significant advantage for laparoscopic treatment in terms of pain in the short (VAS = 0 at one month 31.4% *vs.* 0%) and medium term (VAS = 0 at six months 82.8% *vs.* 80%), days on analgesics (6.8 *vs.* 15.9), length of hospital stay (2.5 *vs.* 5.1 days) and time to resumption of normal activities (14 *vs.* 27 days). Conversely, the differences were not statistically significant for pain at one year (VAS = 0 88.6% *vs.* 90%;) and morbidity (37% *vs.* 40%) [***Confidence in effect estimates: very low due to the observational design of the studies and risk of imprecision***].

**Patient values and preferences.** No evidence was found in the literature. In the outcome prioritization phase, the patient representatives and patient advocate expressed their preferences in line with those of the rest of the panel. For this reason, the panel believed there was no variability or uncertainty about patients' values and preferences.

**Balance between desirable/undesirable effects.** It was impossible to balance desirable and undesirable effects since the evidence did not allow the panel to evaluate them sufficiently and were limited to a single type of border hernia. Therefore, the panel believed that the confidence in the estimates was too low, and a recommendation would have been too speculative.

**Equity, acceptability, and feasibility.** The suggested approach has no equity, acceptability, or feasibility issues in the Italian health system context.

#### Parastomal hernias

**PICO 1F.** In patients with a parastomal hernia (P), is it preferable to choose laparoscopic (I) or open (C) surgery in terms of (O) mortality, morbidity, recurrence, quality of life, length of hospitalization, postoperative pain, and costs?

**RECOMMENDATION 1F.** The panel suggested that laparoscopic surgery be used as an alternative to open treatment for patients with a parastomal hernia.

**STRENGTH OF RECOMMENDATION.** Conditional, based on VERY LOW confidence in effect estimates, deriving from observational studies burdened with risk of bias, indirectness, and imprecision.

**Evidence synthesis.** The literature search retrieved two systematic reviews of randomized and observational studies that compared the laparoscopic and open techniques in patients with parastomal hernias [55, 56].

Two additional observational studies directly compared the outcomes of the two techniques [57, 58]. Halabi et al*.* [57] examined 1,945 patients with parastomal hernia operated by open technique and 222 by laparoscopy, extracted from the ACS-NSQIP database (2005–2011). Keller et al*.* [58] examined 62 patients (31 operated with a laparoscopic and 31 with an open technique). Mortality was examined in the study by Halabi et al*.*: the authors found a mortality rate of 0.45% in the laparoscopic *vs.* 1.59% in the open group (RR 0.28, 95%CI 0.04–2.06) [**Confidence in effect estimates: very low due to risk of bias and indirectness**]. In addition, Halabi et al*.* [57] demonstrated lower rates of overall morbidity in cases treated with laparoscopy, with a 68% reduction in the risk of developing complications (27.04% *vs.* 11.71%, RR 0.32, 95%CI 0.30–0.63), 73% in superficial infections (9.97% *vs.* 2.70%, RR 0.27, 95%CI 0.12–0.60) and 56% in deep surgical site or organ infection (6.12% *vs.* 2.70%, RR 0.44, 95%CI 0.20–0.99) [***Confidence in effect estimates: very low due to risk of bias***].

Keller et al*.* [58] found a lower number of wound dehiscences in the laparoscopic compared to the open group (3% *vs.* 29%; RR 0.11, 95%CI 0.01–0.82). Similarly, the difference in superficial surgical site infection rates was 10% *vs.* 32% (RR 0.30, 95%CI: 0.09–0.99), in wound complications 29% *vs.* 52% (RR 0.56, 95%CI: 0.29–1.07), and in other complications 36% *vs.* 52% (RR 0.69, 95%CI: 0.38–1.23) in favor of the laparoscopic approach [***Confidence in effect estimates: very low due to risk of bias and imprecision***].

The 3-year recurrence rate, assessed with the Kaplan–Meier curves in the study by Keller et al*.* [58], was lower in patients treated by laparoscopy (79% ± SE 9% *vs.* 36% ± SE 15%) [***Confidence in effect estimates: very low due to risk of bias and imprecision***]. In both observational studies, the length of hospitalization was significantly shorter in the laparoscopic group [***Confidence in effect estimates: very low due to risk of bias and imprecision***].

**Patient values and preferences.** No evidence was found in the literature. In the outcome prioritization phase, the patient representatives and patient advocate expressed their preferences in line with those of the rest of the panel. For this reason, the panel believes that there was no variability or uncertainty about patients' values and preferences.

**Balance between desirable/undesirable effects.** Laparoscopic surgery in patients with parastomal hernia proved beneficial for all outcomes examined. The literature did not highlight any undesirable effects. The diameter of the defect already expressed for the PICO 1A must also be considered in this subgroup of patients.

**Equity, acceptability, feasibility.** The suggested approach has no equity, acceptability, or feasibility issues in the Italian health system context.

### RESEARCH QUESTION 2: What is the optimal minimally invasive technique in patients with primary or incisional ventral hernia?

For Question 2, the panel considered the extreme variability of the single clinical conditions and of the different settings in which the intervention could be performed. Therefore, the panel did not intend to define each operative technical step but identified some aspects that appeared controversial or yet to be defined.

The techniques whose relatively recent introduction has yet to allow the production of scientific literature sufficient for a comparison with the most common approaches were not considered (endoscopic retro-muscular positioning of the mesh, laparoscopic or robotic component separation).

#### Suture of the hernia defect: IPOMplus vs. IPOM

**PICO 2A.** In the laparoscopic treatment of patients with a primary or incisional ventral hernia (P), is it preferable to close the defect (IPOM plus) (I) or not (IPOM) (C) in terms of (O) mortality, morbidity, recurrence, quality of life, length of stay, postoperative pain, and costs?

**RECOMMENDATION 2A.** The panel suggested that the hernia defect be sutured in the laparoscopic treatment of patients with a primary or incisional ventral hernia.

**STRENGTH OF RECOMMENDATION.** Conditional, based on VERY LOW confidence in effect estimates. The analysis came from observational studies with a high risk of bias, heterogeneity, and imprecision of the estimates; and from randomized controlled trials with a low risk of bias but with severe imprecision and indirectness.

**Evidence synthesis.** To date, four systematic reviews of randomized trials and observational studies have addressed the issue of hernia defect closure during laparoscopic primary or incisional hernia repair. Three of the four systematic reviews carried out a qualitative analysis only [59–61], and one included a quantitative analysis [62]. Tandon et al*.* [62] included seven non-randomized comparative studies [63–69]. It also included the study by Gonzalez et al*.* [67], which compared a laparoscopic arm with a robotic one, and the study by Chelala et al*.* [69], which included only a limited number of historical comparators prior to the introduction of the defect closure technique. It did not include the studies by Light et al*.* [70], Papageorge et al*.* [71], Martin-Del-Campo et al*.* [72], Nguyen et al*.* [73], Karipineni et al*.* [74], Suwa et al*.* [75], Sadava et al*.* [76] and Baker et al*.* [77], published later. Four randomized controlled trials that compared laparoscopic IPOMplus *vs.* IPOM were retrieved from the literature search [78–81]. In the study by Ahonen-Siirtola et al*.* [78], however, the IPOMplus group consisted of patients operated on with a hybrid technique (open and laparoscopic). A further meta-analysis that included the new three randomized controlled trials was published in 2021, but it contained some inaccuracies [82]. We then performed a new pooled analysis, including all the published studies except those by Gonzales et al*.* [67], Chelala et al*.* [69], and the randomized trial by Ahonen Siirtola et al*.* [78]. The analysis was carried out separately for randomized and observational studies.

**Fig. 5:**
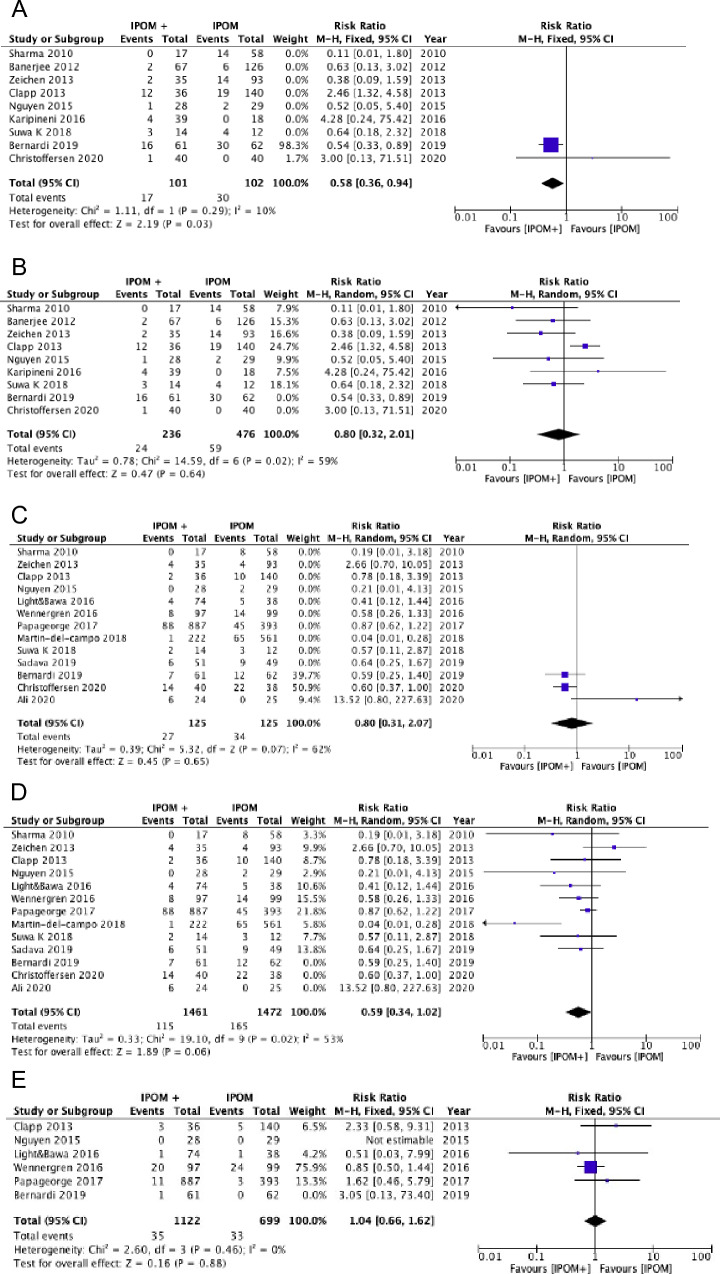
Laparoscopic IPOM vs. IPOMplus: Postoperative morbidity—RCTs (**A**), Postoperative morbidity—Observational studies (**B**), Seromas—RCTs (**C**), Seromas—Observational studies (**D**), Surgical-Site Infections (**E**)

Our meta-analysis of overall morbidity included nine comparative studies (337 *vs.* 578 patients) from two randomized controlled trials, one of which only involved umbilical hernias. Statistically significant differences were not found either in terms of overall morbidity or some of its specific components (16.8% *vs.* 29.4%; RR 0.58; 95%CI 0.36–0.94;* I*^*2*^ = 10%) when analyzing randomized controlled trials only [***Certainty of evidence: very low due to imprecision and indirectness***] and when analyzing seven observational studies (10.1% *vs.* 0.12%; RR 0.80; 95%CI 0.32–2.01; *I*^*2*^ = 59%) [***Certainty of evidence: very low certainty due to high risk of bias, heterogeneity, and imprecision***] (Fig. 5).

**Fig. 6:**
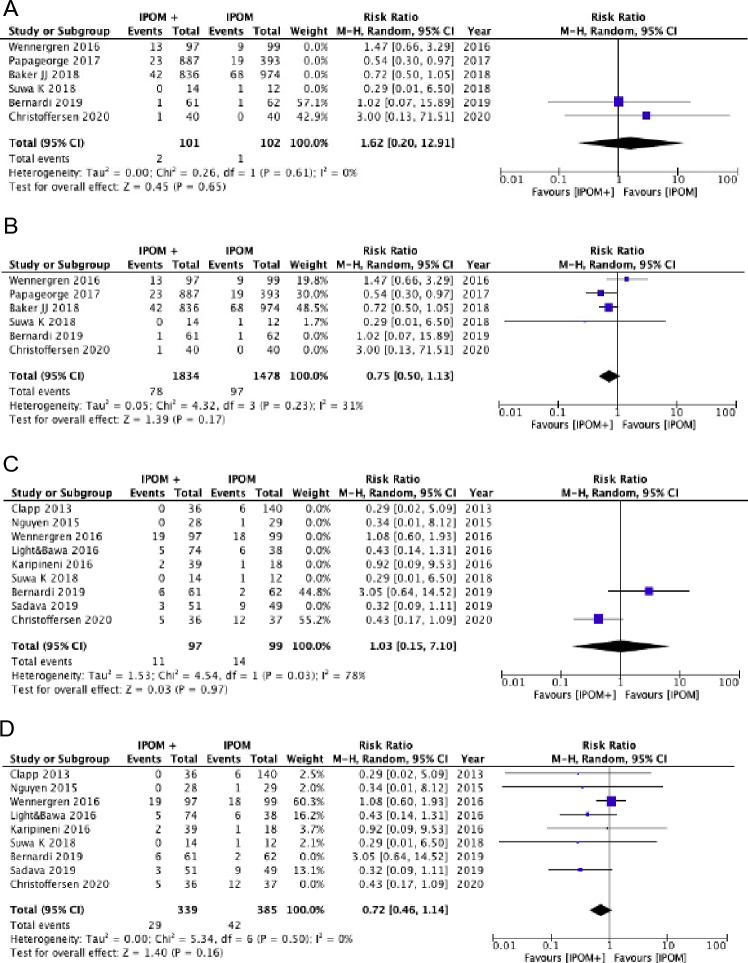
Laparoscopic IPOM vs. IPOMplus: Re-operations—RCTs (**A**), Re-operations—Observational studies (**B**), Hernia recurrence—RCTs (**C**), Hernia recurrence—Observational studies (**D**)

Thirteen studies examined the incidence of seroma. The pooled analysis of the three randomized trials showed no difference between the two techniques (21.6% *vs.* 27.2%; RR 0.80; 95%CI 0.31–2.07; *I*^*2*^ = 65%) [***Certainty of evidence: very low due to imprecision and heterogeneity***]. Similarly, the pooled analysis of the observational studies showed no differences, although a trend in favor of IPOMplus was reported (7.9% *vs.* 11.2%; RR 0.59; 95%CI 0.34–1.02; *I*^*2*^ = 53%) [***Certainty of evidence: very low due to high risk of bias and imprecision***] (Fig. 5). Surgical site infections were analyzed in five observational studies without any difference between the two groups (3.1% *vs.* 4.7%; RR 1.04; 95%CI 0.66–1.62; *I*^*2*^ = 0%) [***Certainty of evidence: very low due to high risk of bias and imprecision***] (Fig. 5). In addition, the number of re-interventions was examined in two randomized controlled trials without any difference between the two groups (0.98% *vs.* 0.99%; RR 1.68; 95%CI: 0.23–12.53; *I*^*2*^ = 0%) [***Certainty of evidence: low due to imprecision***]. Also, the pooled analysis of the four observational studies did not show substantial differences, although a trend in favor of IPOMplus was reported (74.2% *vs.* 6.5%; RR 0.75; 95%CI: 0.50–1.13; *I*^*2*^ = 31%) [***Certainty of evidence: very low due to high risk of bias and imprecision***] (Fig. 6). Nine studies examined the incidence of hernia recurrence (two randomized controlled trials and seven observational studies). Both the pooled analysis of randomized trials (11.3% *vs.* 14.1%; RR 1.03; 95%CI 0.15–7.10; *I*^*2*^ = 78%) [***Certainty of evidence: very low due to imprecision, indirectness, and heterogeneity***] and observational studies (8.5% *vs.* 10.9%; RR 0.72; 95%CI 0.46–1.14; *I*^*2*^ = 0%) showed no differences between the two techniques [***Certainty of evidence: very low due to high risk of bias and imprecision***] (Fig. 6).

Five studies analyzed postoperative pain. The scales used and the different time intervals at which the pain was detected did not allow for a meta-analysis to be carried out. In the study by Clapp et al*.*, postoperative pain was assessed six months after surgery when the patients were asked to express an evaluation of the worst pain they had experienced (mean 2.47 ± 0.46 *vs.* 3.68 ± 0.76) and on the presence of chronic residual pain (9.3% *vs.* 11.4%) [65]. Bernardi et al*.* reported pain at two years on a scale of 1 to 10 (3.4 *vs.* 2.5) [79]. Finally, Ali et al*.* assessed pain at one week (6 *vs.* 3) and one month (1 *vs.* 0) after surgery, while they did not detect the presence of pain at 6 and 12 months [81]. Christoffersen et al*.* used a scale of 1 to 100 to analyzed pain on the first postoperative day (median 73 *vs.* 69), at 30 days (2 *vs.* 2), and at two years (1 *vs.* 2) [80]. Rogmark et al*.* analyzed the area under the curve of the VAS measurements (scale from 1 to 100) at 30-day follow-up and did not detect any difference between the two treatment groups [39].

#### Patient values and preferences

No evidence was found in the literature. In the outcome prioritization phase, the patient representatives and patient advocate expressed their preferences in line with those of the rest of the panel. For this reason, the panel believed that there was no variability or uncertainty about patients' values and preferences.

#### Balance between desirable/undesirable effects

The panel considered the balance of effects in favor of IPOMplus due to a low magnitude of desirable effects but the absence of undesirable effects in the literature.

**Costs.** Neither evidence on the use of resources nor cost-effectiveness studies of IPOMplus have emerged in the literature. However, the panel believed that the additional cost of IPOMplus was irrelevant. Although a high cost due to the longer average duration of the intervention with IPOMplus (IPOMplus: 68–139 min; IPOM: 60–95), which translates into more extended hours of occupation of the operating room, there were fewer days of hospital stay with IPOMplus. These cost factors were equivalent, thus not translating into additional costs for IPOMplus.

**Equity, acceptability, and feasibility.** The suggested approach has no equity issues in the Italian health system context. Regarding acceptability, the panel believed there might be some variability among the stakeholders. In particular, surgeons without adequate technical skills may not agree to practice IPOMplus. Although IPOMplus requires ad-hoc training, the panel believes that IPOMplus is feasible.

**Note to the recommendation.** The panel issued a recommendation in favor of IPOMplus conditioned not only by the very low certainty of the effects but also by the size of the defect to be sutured and the skill (training, learning curve) of the surgeon. The panel considered the modest dimensions (average between 3 and 5 cm) of the defects included in the studies. The characteristics of the defect (diameter, physical characteristics of the wall) can make it difficult and sometimes impossible to suture the defect.

#### Robotic versus laparoscopic technique

**PICO 2B.** In patients undergoing minimally invasive surgery for ventral or incisional hernia (P), is a robotic (I) or laparoscopic (C) approach preferable in terms of (O) mortality, morbidity, recurrence, quality of life, length of stay, postoperative pain, costs?

**RECOMMENDATION 2B.** For patients with a primary or incisional ventral hernia, the panel suggested that either laparoscopic or robotic techniques be used.

**STRENGTH OF RECOMMENDATION.** Conditional, based on LOW confidence in effect estimates, deriving from meta-analyses of randomized controlled trials and propensity score-matched studies, some of which have a high risk of bias.

**Fig. 7:**
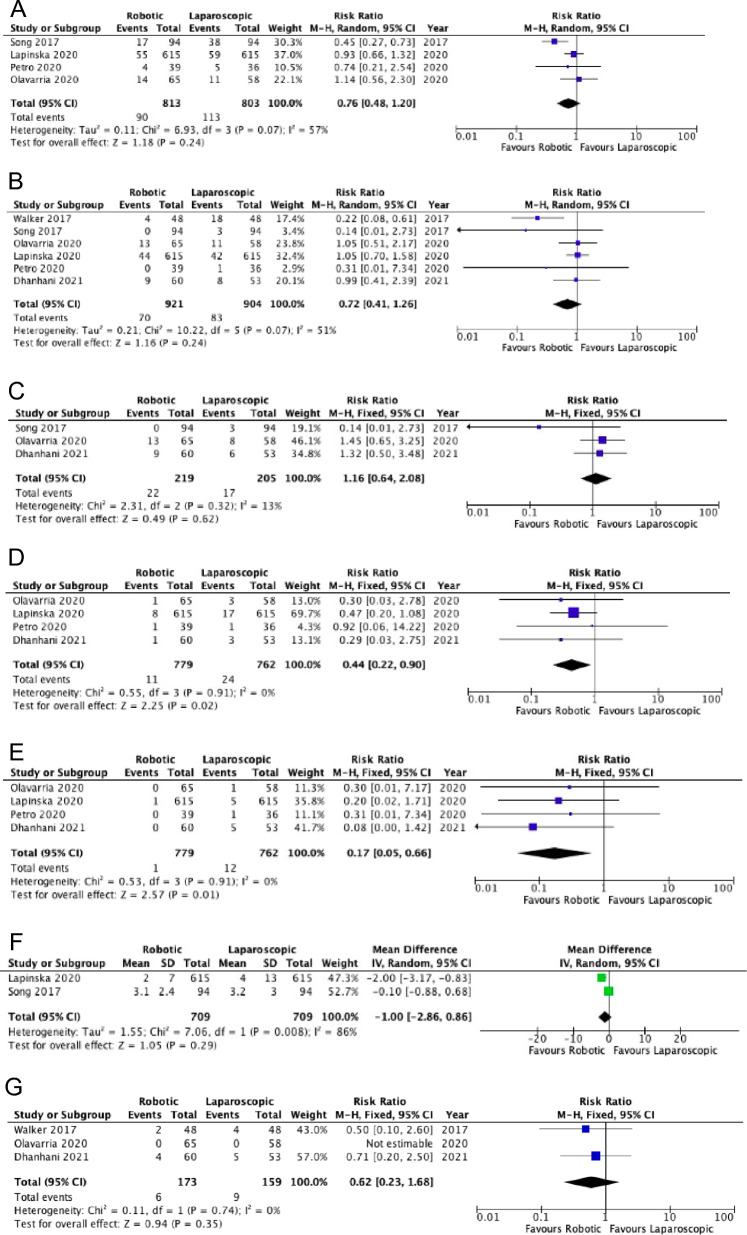
Robotic vs. Laparoscopic repair: Postoperative morbidity (**A**), Surgical-Site Occurrence (**B**), Seroma (**C**), Hospital readmission (**D**), Re-operation (**E**), Length of hospital stay (**F**), Recurrence (**G**)

**Evidence synthesis.** The comparison between robotic and laparoscopic techniques was examined in a recent systematic review and meta-analysis of randomized and observational propensity score-matched studies [83], and another of only observational studies [84]. A new observational study including 679 patients operated with the robotic technique and 20,896 patients operated by laparoscopy was recently published by Altieri et al*.* [85]. However, it showed a high risk of bias due to the unequal baseline distribution of comorbid factors and confounding between the two groups. The meta-analysis by Mohan et al*.* included six studies with a total of 1,959 patients. In three of the six studies (propensity score-matched), however, the technique adopted (IPOM or retro muscular positioning) was not specified, whereas one of the studies considered only a population of patients with obesity [83]. Furthermore, the meta-analysis report was burdened with numerous formal errors and did not consider some outcomes of interest. We, therefore, decided to perform a new meta-analysis of randomized controlled and propensity score-matched studies. No differences emerged between the robotic and laparoscopic techniques for overall morbidity (11.07% *vs.* 14.07%; RR 0.76, 95%CI 0.48–1.20;* I*^*2*^ = 57%); surgical site occurrence (7.6% *vs.* 9.2%; RR 0.72, 95%CI 0.41–1.26; *I*^*2*^ = 51%); or seroma (10.5% *vs.* 8.5%; RR 1.16, 95%CI 0.64–2.08; *I*^*2*^ = 13%). However, we found a difference in favor of the robotic technique in the number of hospital re-admissions at 30 days (1.4% *vs.* 3.1%; RR 0.44, 95%CI 0.22–0.90; *I*^*2*^ = 0%) and re-interventions (0.1% vs. 1.5%; RR 0.17, 95%CI 0.05–0.66; *I*^*2*^ = 0%) (Fig. 7) [***Certainty of evidence: low due to high risk of bias***].

The trend in overall morbidity was confirmed in the study by Henricksen et al*.* [84] (robotic *vs.* laparoscopic techniques: 14% *vs.* 16%; OR 0.60, 95%CI 0.20–1.75; *I*^*2*^ = 88%) [***Certainty of evidence: very low due to high risk of bias and heterogeneity***].

Conversely, in the study by Altieri et al*.* [85], the univariate analysis showed worse outcomes for patients in the robotic group concerning overall morbidity (20.21% *vs.* 10.56%), hospital re-admissions (9.28% *vs.* 5.06%), length of hospital stay (4.32 ± 18 *vs.* 2.19 ± 6.3 days) and unplanned access to the emergency department at 30-days follow-up (14.4% *vs.* 10.5%). However, the authors also performed a propensity score-matched case–control analysis showing an overall morbidity difference in favor of the robotic group (risk difference –0.0575; 95%CI: –0.1023 to –0.0128) [***Certainty of evidence: very low certainty due to high risk of bias***].

Our meta-analysis did not reveal a difference in hernia recurrence rates between the two approaches, although a trend in favor of robotics was evident (7.6% *vs.* 9.2%; RR 0.62; 95%CI 0.23–1.68; *I*^*2*^ = 0%) [***Certainty of evidence: low certainty due to high risk of bias***] (Fig. 7).

The meta-analysis of observational studies published by Henriksen et al*.* [84] included four studies that compared robotic IPOM *vs.* laparoscopic IPOM [67, 86, 87], a study that compared a robotic retro muscular with a laparoscopic intraperitoneal technique[88] and two registry studies in which it was not possible to assess what robotic techniques were adopted [41, 89]. One of the four included studies compared a robotic technique with defect closure (rIPOMplus) with a laparoscopic technique without defect closure (lapIPOM) [67]. Therefore, that meta-analysis was burdened by high heterogeneity.

Our meta-analysis included two studies investigating the difference in mean hospital stay between the laparoscopic and robotic approaches (1,418 patients). A difference between the two techniques was not demonstrated (MD − 1.00; 95% CI − 2.86–0.86; *I*^*2*^ = 86%) (Fig. 7) [***Certainty of evidence: very low due to high risk of bias and heterogeneity***]. The meta-analysis by Henriksen et al*.* [84] included four studies analyzing the length of hospital stay. The pooled analysis showed a statistically significant difference in favor of the laparoscopic technique (MD 0.61 days, 95%CI 0.52–0.70). The limitation related to the high risk of bias of the study by Altieri et al*.* [85] also applied to the hospital stay, longer in the robotic group both in the non-matched (4.32 ± 18 *vs.* 2.19 ± 6.3 days, P = 0.0023), and matched analyses (MD 1 day) [***Certainty of evidence: very low due to high risk of bias***].

**Costs.** The systematic review of the literature did not retrieve cost–benefit or cost-effectiveness studies. Therefore, it was not possible to perform a complete economic assessment. However, higher costs for the robotic group were reported in the meta-analyses by Henricksen et al*.* [84] and Mohan et al*.* [90].

**Patient values and preferences.** No evidence was found in the literature. In the outcome prioritization phase, the patient representatives and patient advocate expressed their preferences in line with those of the rest of the panel. For this reason, the panel believed there was no variability or uncertainty about patients' values and preferences.

**Balance between desirable/undesirable effects.** The robotic technique's lower morbidity (overall, local complications, re-operations, and hospital readmission) was a desirable effect. Costs cannot be thoroughly analyzed without a complete economic evaluation that related costs and outcomes. The panel evaluated the balance between desirable and undesirable effects in favor of the robotic approach.

**Equity, acceptability, and feasibility.** The limited diffusion of robotic technology on the national territory must be taken into account, as well as its concentration mainly in large centers. The limited availability affects both equity (patients far from large centers have fewer possibilities to have access to robotics) and feasibility (robotic intervention may not be feasible due to the absence of the technology or the limitation of its use to other clinical conditions considered to be of highest priority in the use of the resource). The suggested approach has no acceptability issues in the Italian health system context.

#### Laparoscopic surgical technique for parastomal hernia

**PICO 2C.** In patients undergoing laparoscopic parastomal hernia repair (P), is the Keyhole (I) or Sugarbaker (C) technique preferable in terms of (O) mortality, morbidity, recurrence, quality of life, length of stay, postoperative pain, and costs?

**RECOMMENDATION 2C.** For the laparoscopic treatment of parastomal hernias, the panel suggested that the Sugarbaker technique be used rather than the keyhole technique.

**STRENGTH OF RECOMMENDATION.** Conditional, based on VERY LOW confidence in effect estimates. The data derived from observational studies with a high risk of bias, indirectness, and imprecision.

**Evidence synthesis.** From the literature search, three systematic reviews and meta-analyses were retrieved, including 17 non-randomized observational studies that described the application of the laparoscopic technique in treating patients with a parastomal hernia (ileostomy and colostomy) [90–93]. Almost all the studies considered in the meta-analyses presented case series of patients treated with the laparoscopic Sugarbaker and keyhole techniques. In addition, DeAsis et al*.* and Hansson et al*.* also included a series (n = 47 patients) of the so-called "sandwich technique" [56, 93]. The three meta-analyses directly compared Sugarbaker versus keyhole techniques and focused on recurrence. For the other postoperative outcomes (overall morbidity, complications, hospital stay, and mortality), the specific results for each technique were not always extractable for the single approaches.

In the analysis of DeAsis et al*.* [93], the global mortality rate was 1.8% (range 0.8–3.2), while Hansson et al*.* [56] reported a rate of 1.2% (range 0.3–3.0). These results included Sugarbaker and keyhole laparoscopic techniques [***Certainty of evidence: very low certainty of evidence due to risk of bias and indirectness***]. DeAsis et al*.* [93] reported an overall postoperative complication rate after laparoscopic repair of parastomal hernias of 1.8% (range 0.8–3.2), with a 1.7% (range 0.7–3.1) mesh infection rate, 3.8% (range 2.3–5.7) rate of surgical site infection, and 1.7% (range 0.7–3.0) intestinal obstruction. Similar results were reported by Hansson et al*.* [57] with a 3.3% (range 1.6–5.7) risk of wound infection and a risk of mesh infection of 2.7% (range 1.2–5) [***Certainty of evidence: very low certainty of evidence due to risk of bias and indirectness***].

The three meta-analyses highlighted that the Sugarbaker technique was associated with a lower incidence of recurrence than the keyhole, suggesting that applying an intraperitoneal mesh with parietalization of the bowel reduced the risk of failure. In particular, Knaapen et al*.* [55] reported a recurrence rate of the Sugarbaker technique versus the keyhole of 10.9% (95%CI 3.7–21.4) *vs.* 35.6% (95%CI 14.6–60.1), respectively (OR 0.35; 95%CI 0.21–0.59). DeAsis et al*.* [93] also described a reduction in recurrence in favor of the Sugarbaker technique (10.2% *vs.* 27.9%), in keeping with what was found by Hansson et al*.* [56] who reported an incidence of 11.6% (95%CI 6.4–18.0) *vs.* 20.8% (95%CI 15.0–27.3) [***Certainty of evidence: very low due to risk of bias, indirectness and imprecision***].

**Patient values and preferences.** No evidence was found in the literature. In the outcome prioritization phase, the patient representatives and patient advocate expressed their preferences in line with those of the rest of the panel. For this reason, the panel believed there was no variability or uncertainty about patients' values and preferences.

**Balance between desirable/undesirable effects.** The Sugarbaker technique had a reduced recurrence rate compared to the keyhole technique. The literature did not compare mortality and morbidity outcomes between the two techniques. The panel evaluated the balance between effects desirable and undesirable in favor of the Sugarbaker technique.

**Equity, acceptability, and feasibility.** The suggested approach has no equity, acceptability, or feasibility issues in the Italian health system context.

#### Transfixed sutures vs. tacks

**PICO 2D.** In the laparoscopic repair of patients with a primary or incisional hernia (P), is it preferable to fix the mesh with sutures (I) or mechanical tacks (C) in terms of (O) mortality, morbidity, recurrence, quality of life, length of stay, postoperative pain, and costs?

**RECOMMENDATION 2D.** In the laparoscopic treatment of patients with a primary or incisional ventral hernia, the panel suggested that the mesh be fixed by mechanical tacks rather than transfixed sutures.

**STRENGTH OF RECOMMENDATION.** Conditional, based on VERY LOW confidence in effect estimates, deriving from meta-analyses of randomized controlled trials, which, however, presented a high or uncertain risk of bias, imprecision, and indirectness. The risk of publication bias cannot be assessed.

**Evidence synthesis.** Two meta-analyses of randomized trials [59, 60], one of observational studies [61] and one including randomized and non-randomized studies [94], examined the differences between mechanical and suture fixation of the mesh. The randomized studies included in the meta-analyses by Sajid et al*.* [94] and Baker et al*.* [91] were included in that by Ahmed et al*.* [90], which was also the most recent and best-rated meta-analysis according to the AMSTAR II criteria. It included five randomized trials [95–99]. However, this meta-analysis compared metallic tacks with sutures but included heterogeneous methods for mesh fixation. Beldi et al*.* [95] directly compared non-absorbable sutures to metal tacks. Bansal et al*.* [98] included mesh fixation with permanent sutures in both arms, one of which also employed metal tacks. In contrast, Muysoms et al*.* [99] and Wassenaar et al*.* [97] compared metallic tacks fixation with a combination of metal tacks and sutures.

During the preparatory work for this guideline, Mathes et al*.* published a Cochrane review about different fixation techniques [100]. There were neither differences in pain measured with a VAS 4–6 weeks after surgery (MD 0.18; 95%CI 0.48–0.85;* I*^*2*^ = 90%) nor in the incidence of chronic pain at 3–6 months (OR 1.24; 95%CI 0.65–2.38; *I*^*2*^ = 27%) [***Certainty of evidence: very low due to high risk of bias, indirectness, and heterogeneity***].

The meta-analysis by Mathes et al*.* [100]. also found no differences for either chronic or acute pain between the two techniques (metallic fixation *vs.* sutures) separately or in combination. Individual comparisons, however, included only one or two studies [***Certainty of evidence: very low certainty of evidence due to high risk of bias, and imprecision***].

The meta-analysis by Ahmed et al*.* [90] revealed no differences between the two fixation techniques in terms of recurrence (OR 1.11; 95%CI 0.34–3.62) [***Certainty of evidence: very low due to high risk of bias and indirectness***]. Similarly, Reynvoet et al*.* [92] revealed no differences in the recurrence rate between the two methods [***Certainty of evidence: very low certainty of evidence due to risk of bias***].

Regarding the duration of surgery, the meta-analysis by Ahmed et al*.* [90] reported a difference in favor of the metal tacks fixation group (MD − 19.25; 95%CI − 27.98 to − 10.51) [***Certainty of evidence: very low certainty of evidence due to risk of bias and indirectness***]. The meta-analysis by Mathes et al*.* [100] also found a difference in favor of mechanical tacks when used alone but in favor of sutures alone when compared with combinations of mechanical tacks plus sutures [***Certainty of evidence: very low due to high risk of bias and imprecision***].

The meta-analysis by Ahmed et al*.* [100] revealed no differences either for the length of hospital stay (MD − 0.06; 95%CI − 0.19–0.08;* I*^*2*^ = 0%) and for the occurrence of seroma and hematoma (OR 0.60; 95%CI 0.29–1.26; *I*^*2*^ = 0%) [***Certainty of evidence: very low due to high risk of bias and indirectness***].

**Patient values and preferences.** No evidence was found in the literature. In the outcome prioritization phase, the patient representatives and patient advocate expressed their preferences in line with those of the rest of the panel. For this reason, the panel believed there was no variability or uncertainty about patients' values and preferences.

**Balance between desirable/undesirable effects.** The use of mechanical tacks for the fixation of the mesh was beneficial for the reduction of the operative time. No other desirable effects were noted except for a tendency to have less seroma and hematoma formation. The panel evaluated the balance between desirable and undesirable effects in favor of fixing by mechanical means.

**Equity, acceptability, and feasibility.** The suggested approach has no equity, acceptability, or feasibility issues in the Italian health system context.

#### Absorbable tacks vs. non-absorbable tacks

**PICO 2E.** For the laparoscopic treatment of patients with a ventral or incisional hernia (P), is it preferable to fix the mesh with absorbable (I) or non-absorbable (C) fixation devices in terms of (O) mortality, morbidity, recurrence, quality of life, length of stay, postoperative pain, and costs?

**RECOMMENDATION 2E.** For the laparoscopic treatment of patients with a primary or incisional ventral hernia, the panel suggested that the mesh be fixed either with absorbable or permanent devices.

**STRENGTH OF RECOMMENDATION.** Conditional, based on VERY LOW confidence in effect estimates, deriving from meta-analyses of randomized controlled trials, some of which presented a high or uncertain risk of bias and imprecision.

**Evidence synthesis.** The use of absorbable tacks as a method of mesh fixation was introduced to reduce chronic postoperative pain. On the other hand, it was hypothesized that using absorbable tacks could favor recurrence. Two systematic reviews and meta-analyses considered the comparison between absorbable and permanent tacks. The first, published by Khan et al*.* in 2018 [101], included three randomized controlled trials [97, 102, 103] and two observational studies [104, 105] for a total of 1,149 patients. One of the included randomized trials did not compare directly absorbable and metallic tacks but different combinations of fixation methods, including metallic tacks [96]. For this reason, this meta-analysis was not considered in our review. The second meta-analysis included two randomized controlled trials [103, 106] and was taken as a reference [100]. The study by Colak et al*.* [103] showed a high risk of bias (performance, detection and attrition bias). The trial by Harslof et al*.* [106] had a follow-up of only 12 months (indirectness), and 20% of patients were lost to follow-up in one group. The meta-analysis by Mathes et al*.* [100] demonstrated no differences between absorbable and permanent tacks in terms of recurrence (RR 0.74, 95%CI 0.17–3.22) [***Certainty of evidence: very low for high risk of bias and imprecision***].

Postoperative pain was analyzed separately for the two studies included in the meta-analysis [100].

Colak et al*.* [103] evaluated postoperative pain with a VAS 0–100 scale (at two days: MD − 11.80, 95%CI − 27.71–4.11; two weeks: MD 0.40 95%CI − 0.01–0.81; six months: MD 0.50 95%CI − 0.08–1.08) [***Certainty of evidence: very low for high risk of bias and imprecision***]. Harslof et al*.* [106], pain assessment was performed with the Dolo™ Test questionnaire and showed no difference between absorbable and non-absorbable tacks (55.3 ± 28.9 *vs.* 43.5 ± 28.5 on a scale from 0 to 100) [***Certainty of evidence: low certainty of evidence due to indirectness and high risk of bias***].

**Patient values and preferences.** No evidence was found in the literature. In the outcome prioritization phase, the patient representatives and patient advocate expressed their preferences in line with those of the rest of the panel. For this reason, the panel believed there was no variability or uncertainty about patients' values and preferences.

**Balance between desirable/undesirable effects.** From the direct comparison between the use of these two modes of fixation, no differences emerged for any of the outcomes examined, particularly the incidence of postoperative pain (acute or chronic) and recurrences.

**Equity, acceptability, feasibility.** The suggested approach has no equity, acceptability, or feasibility issues in the Italian health system context.

#### Mesh overlap

**PICO 2F.** For the laparoscopic treatment of patients with a ventral or incisional hernia (P) with the IPOM technique, what is the optimal overlap of the mesh on the abdominal wall surface (I) (C) in terms of (O) mortality, morbidity, recurrence, quality of life, length of stay, postoperative pain, and costs?

**RECOMMENDATION 2Fa.** In treating patients with a primary or incisional ventral hernia with a defect diameter of 4 cm or larger, the panel suggested a minimum overlap of the mesh beyond the margins of the defect of 5 cm on each side.

**STRENGTH OF RECOMMENDATION.** Conditional, based on VERY LOW confidence in effect estimates, deriving from observational studies with a high risk of bias.

**RECOMMENDATION 2Fb.** In the treatment of patients with primary or incisional ventral hernia, with a defect less than 4 cm, the panel suggested a minimum overlap of the mesh beyond the margins of the defect of 3–5 cm on each side.

**STRENGTH OF RECOMMENDATION.** Conditional based on VERY LOW confidence in effect estimates, deriving from observational studies with a high risk of bias.

**Evidence synthesis.** A systematic review of the literature analyzed the amount of overlap between the mesh and the abdominal wall necessary to decrease the recurrence rate in laparoscopic IPOM [107]. After this systematic review, Hauters et al*.* [108] published an observational study with uni- and multivariate analyses on 213 patients with a median follow-up of 69 ± 44 months. However, this single-center study was characterized by numerous patients with a hernia defect diameter inferior to 2 cm (62%) and a high recurrence rate in patients with a defect larger than 2 and 4 cm (14.8% and 26.9%, respectively). The systematic review by LeBlanc et al*.* [106] included randomized trials and observational studies. It stratified the results into three groups (overlap < 3 cm, 3–5 cm, and > 5 cm) and showed that, in laparoscopic IPOM, the risk of recurrence decreased with the increase of the overlap area, being minimal when an overlap of at least 5 cm was guaranteed (incidence of recurrence was 8.6%, 4.6%, and 1.4%, respectively). The authors also performed a subgroup analysis according to the size of the defect (< 4 cm, 4–10 cm, > 10 cm) and found that, in patients with a defect diameter greater than 4 cm, the recurrence rate decreased from 7 to 3% when a minimum overlap of 5 cm was granted. For defects less than 4 cm, no studies with an overlap > 5 cm were available; however, the recurrence rate was halved when an overlap between 3 and 5 cm was applied on each side. Confidence in the results of this review was limited by the high risk of bias (absence of control groups in most of the studies considered) and uncertainty about the direct transferability of the data to the daily practice (variable follow-up of the included studies) [***Certainty of evidence: very low due to indirectness and high risk of bias***].

While keeping in mind its relevant limitations (high risk of bias and indirectness), the study by Hauters et al*.* [108] confirmed that an overlap of less than 5 cm was a predictor of recurrence in the univariate analysis. However, the only predictive factor of hernia recurrence in the multivariate analysis was the ratio between the mesh area and that of the defect (coefficient − 0.79, OR 0.46, 95%CI 0.276–0.741). For ratios of < 8, 9–12,13–16, and ≥ 17, the recurrence rate was 70%, 35%, 9%, and 0%, respectively, highlighting that the length of the overlap cannot be standard but must be related to the area of the defect [109, 110] [***Certainty of evidence: very low certainty of evidence due to high risk of bias and indirectness***].

**Patient values and preferences.** No evidence was found in the literature. In the outcome prioritization phase, the patient representatives and patient advocate expressed their preferences in line with those of the rest of the panel. For this reason, the panel believed there was no variability or uncertainty about patients' values and preferences.

**Balance between desirable/undesirable effects.** Although the ideal minimal overlap of the mesh to the abdominal wall appears to vary with the defect area, most studies measured the overlap in centimeters beyond the edge of the hernia defect. Therefore, in the balance between desirable and undesirable effects and the recommendation formulation, it was necessary to consider the data expressed with this second parameter. The desirable effect of a reduction in hernia recurrence was obtained with a minimum overlap of 5 cm beyond the edge of the defect. However, no studies considered such overlap for defects smaller than 4 cm in diameter. In this case, a reduction in recurrences could be demonstrated with a minimum overlap between 3 and 5 cm on each side. The panel, therefore, considered that the balance between desirable and undesirable effects favors a minimum overlap of 5 cm for defects greater than 4 cm and between 3 and 5 cm for smaller defects.

**Equity, acceptability, feasibility.** The suggested approach has no equity, acceptability, or feasibility issues in the Italian health system context.

**Note to the recommendation.** The panel produced recommendations on the extent of mesh overlap to the hernia defect conditioned, besides the very low certainty of the effects, also by the defect size. In the individual case, the choice must also consider that the extent of the overlap could be better represented by the ratio between the area of the defect and that of the mesh rather than a linear measure in centimeters. The panel considered this aspect but noted that the current literature mainly adopts a linear measure.

### RESEARCH QUESTION 3: What is the optimal depth of the neuromuscular block (deep vs. intermediate) in patients with primary or incisional ventral hernia?

#### Deep vs. moderate neuromuscular block

**PICO 3.** In patients operated with a minimally invasive approach for primary or incisional ventral hernia, or parastomal hernia (P), is a deep neuromuscular block (I) preferable to a moderate one (C) (TOF > 0), in terms of (O) success of the procedure, evaluation of the intra-abdominal workspace, postoperative pain, operator satisfaction, and difficulty of the procedure?

**RECOMMENDATION 3.** The panel could not issue any recommendation in favor or against a deep neuromuscular block.

**STRENGTH OF RECOMMENDATION.** No recommendation. The confidence in effect estimates in this research field was VERY LOW, as it derived from a single randomized controlled study burdened by imprecision, indirectness, and high risk of bias.

**Evidence synthesis.** The only comparative study about this research question was a randomized, crossover (deep neuromuscular block—administered in the first or second phase of the intervention alternately in the two groups), assessor-blinded, controlled trial. Its sample size was small (34 patients), and its primary outcome was the difference in the vision of the operative field between a deep neuromuscular block (NMB) and no block rather than an intermediate block (indirectness) [111]. The outcome was judged by the surgical staff on a 5-level scale (0 = "extremely poor conditions"; 5 = "optimal conditions "). Secondary outcomes were the evaluation of the intra-abdominal operating space after the second administration at the time of suturing the hernia defect (evaluated with the judgment "improved", "unchanged", or "worsened ") and operating time. However, the outcome assessment was burdened by an uncertain risk of performance and detection bias [***Confidence in effect estimates: very low due to imprecision, indirectness, and uncertain risk of bias***].

No difference was found between a deep and no neuromuscular block (MD − 0.1; 95%CI − 0.4–0.2), and in the difference between the first and second phase (no NMB followed by deep NMB, 84% were "unchanged" and 16% "improved"; deep NMB followed by no NMB 30% were "worsened", 40% "unchanged", and 30% "improved"). No difference was found in total operative time (mean: 61 ± 24 min *vs.* 64 ± 31 min) and suturing of the defect (mean: 10 ± 9 min *vs.* 9 ± 7 min). The surgeon's assessment at the defect closure showed a difference in favor of the deep neuromuscular block (mean score 4.8 ± 0.4 *vs.* 4.0 ± 1.4).

**Patient values and preferences.** No evidence was found in the literature. In the outcome prioritization phase, the patient representatives and patient advocate expressed their preferences in line with those of the rest of the panel. For this reason, the panel believed there was no variability or uncertainty about patients' values and preferences.

**Balance between desirable/undesirable effects.** Due to the lack of difference in most of the outcomes considered in the only study available, and also taking into account the absence of direct transferability of the results and scarcity of data (imprecision), the panel believed that the confidence in the estimates was too low, and a recommendation could have been speculative. Therefore, in clinical practice, it will be possible to refer to neuromuscular block guidelines not specific to the treatment of incisional and primary ventral hernias.

**Equity, acceptability, feasibility.** The suggested approach has no equity, acceptability, or feasibility issues in the Italian health system context.

**Note to the recommendation.** In the absence of a recommendation, it is desirable to monitor the neuromuscular block to administer a continuous infusion or multiple doses of muscle relaxant. Furthermore, monitoring is mandatory when surgery is conducted with a deep block (TOF = 0, PTC = 1- 2) and in patients with severe liver or kidney insufficiency or neuromuscular pathology [112].

### RESEARCH QUESTION 4: Should analgesic loco-regional anesthesia be combined with general anesthesia for postoperative pain control in patients with a primary or incisional ventral hernia?

#### Combination of analgesic loco-regional anesthesia with general anesthesia

**PICO 4.** In patients operated on for primary or incisional ventral or parastomal hernia with a minimally invasive approach (P), is it preferable to combine (I) or not to combine (C) analgesic loco-regional anesthesia with general anesthesia in terms of (O) success of the procedure, evaluation of the intra-abdominal workspace, postoperative pain, operator satisfaction, and difficulty of the procedure?

**RECOMMENDATION 4.** In the laparoscopic treatment of patients with a primary or incisional ventral hernia, the panel suggested that regional anesthesia be associated with general anesthesia.

**STRENGTH OF RECOMMENDATION.** Conditional to MODERATE confidence in effect estimates, deriving from randomized controlled trials with a low sample size (imprecision).

**Evidence synthesis**. All the studies focused on a single modality of regional anesthesia (TAP block). The literature search retrieved four randomized studies [113–116] and one economic evaluation study [117]. Two randomized trials compared a group of patients managed with TAP block with bupivacaine performed under laparoscopic guidance with a second group in which a physiological solution was administered with a similar technique in one trial [114] and a placebo in the other [113]. The trials by Sinha et al*.* [115] and Jain et al*.* [116] compared the ultrasound-guided administration of ropivacaine *vs.* placebo. The economic analysis performed by Colonna et al*.* [117] was a cost-utility study of the TAP block with liposomal bupivacaine *vs.* common opioids.

Although with a small sample size (10 *vs.* 10 patients), the study by Ahmed et al*.* [113] demonstrated a reduction in the postoperative use of patient-managed infusion opioids on the first postoperative day (mean administration in mg of morphine sulfate: 22.4 vs. 62.5) favoring the TAP block technique. However, this study was at high risk of bias (absence of staff concealment both at allocation and evaluation of the result and absence of control of confounding factors) [***Confidence in effect estimates: low due to high risk of bias and imprecision***].

Fields et al*.* [115] compared 52 patients undergoing laparoscopic ventral hernia repair with TAP block with bupivacaine with 48 controls. The TAP block was associated with an advantage in terms of lower pain intensity in the immediate postoperative period (1 h after surgery) on a scale from 1 to 10 [average at rest: 5.19 ± 0.39 *vs.* 6.46 ± 0.38; in motion: 6.15 ± 0.42 *vs.* 7.73 ± 0.40 [***Confidence in effect estimates: high***] and lower opioid use (40% reduction at 24 h: 25.64 mg *vs.* 42.56 mg) [***Confidence in effect estimates: high***] compared with administration of saline. Conversely, no difference was found in the intensity of pain measured at 24 h (average at rest: 4.60 ± 0.39 *vs.* 4.52 ± 0.31; in motion: 6.75 ± 0.38 *vs.* 6.98 ± 0.40) [***Confidence in effect estimates: moderate due to imprecision***]. The randomized trial by Sinha et al*.* [115] included 15 patients per group and confirmed the reduction in pain (measured with a 10-level scale) at all measurement cut-offs and, in particular, at the time of the first mobilization (5.3 ± 0.5 *vs.* 7.4 ± 0.8), and discharge (7.5 ± 0.9 *vs.* 8.9 ± 0.6) [***Confidence in effect estimates: moderate due to imprecision***]. The fourth study [116], whose groups included 25 patients each, also confirmed significant differences in all measurements (12 h: 4.24 ± 0.89 *vs.* 2.84 ± 1.21; 24 h: 3.40 ± 0.57 *vs.* 2.28 ± 1.13) [***Confidence in effect estimates: moderate due to imprecision***]. There was also a difference in favor of the TAP block regarding the percentage of patients able to walk at 12 h (0% *vs.* 28%) and 24 h (28 *vs.* 65%) after the operation [***Confidence in effect estimates: moderate due to imprecision***]. The economic analysis showed that the model in which the TAP block was used turned out to be the "dominant" strategy because it simultaneously obtained better results (improvement of QALYs of 0.1) and lower costs (savings of $ 457) [117].

**Patient values and preferences.** No evidence was found in the literature. In the outcome prioritization phase, the patient representatives and patient advocate expressed their preferences in line with those of the rest of the panel. For this reason, the panel believed there was no variability or uncertainty about patients' values and preferences.

**Balance between desirable/undesirable effects.** Along with the intervention's desirable effects, the studies examined did not describe procedural complications of loco-regional analgesia. Therefore, the balance was in favor of the desirable effects. However, it must be emphasized that all the studies assessed only one modality of loco-regional anesthesia (TAP block), which is not the only one available to manage pain in abdominal wall surgery.

**Equity, acceptability, feasibility.** The suggested approach has no equity or acceptability issues in the Italian health system context. However, regarding feasibility, the panel believed there might be some variability among the stakeholders. In particular, the choice of one loco-regional technique rather than another may depend on the specific technical skills of the anesthesiologist and on the availability, in the operating room, of an ultrasonographer to allow the procedure.

**Note to the recommendation.** The panel issued a recommendation conditioned, besides moderate certainty of the effects, on the characteristics of the patient, availability of the equipment to perform the procedure, and the specialist's skills (training, learning curve). [118–120].

## Discussion

This is the first general surgery guideline published on the Italian national guideline database (Sistema Nazionale Linee Guida—SNLG) held by the ISS [6]. In 2017, a new Italian regulation established that the professionals involved in patient care must apply the recommendations in the guidelines approved and published in the database, taking into account the specificity of the single situation. In other words, the clinician must apply the guideline indications unless specific clinical or logistic factors suggest a different course of action. The GRADE methodology, advocated by the ISS for this purpose, is particularly apt to support clinicians in those decisions: the distinction between strong and weak (also termed discretionary, conditional, or qualified) recommendations relies upon uniformity of choices in the decision-making process [121]**.** A panel should issue a strong recommendation when it is likely that almost all informed people would make the recommended choice, with low variability. In contrast, a conditional recommendation implies a more significant impact of individual factors, preferences, and values on the decision.

In our guidelines, all the recommendations are conditional. In fact, the panel agreed that the extreme variability of the possible clinical scenarios (dimension and location of the defect, age, general conditions of the patients, associated diseases, setting, resources, technical and logistic conditions), and the generally low confidence in the effect estimates obtained by the literature, allowed broad discretion in patient and provider choices. It acknowledged that different choices are appropriate for different patients and situations, pointing out to a tailored approach in each case. Most people will still abide by the suggested course of action; however, the decision could be different but still sound in many cases.

Many of the controversies about the role of minimally invasive techniques in the treatment of ventral hernias rely upon assumptions (e.g., the risks of an intra-peritoneal placement of the prosthesis, the need to reconstruct the linea alba and its functional implications) with solid physio-pathological basis, but very difficult to prove in a clinical setting. One of the main challenges for the panel was to remain committed to the literature clinical evidence (or absence of evidence) about them without disregarding the legitimate worries of many surgeons. Once again, the GRADE methodology was appropriate for this purpose: its Evidence-to-Decision process insists that the recommendations, based on the literature evidence, take into account values, preferences, and acceptability by the concerned parties. The involvement of patient representatives and a patient advocate in the multidisciplinary and multi-professional panel proved functional in this process.

The recommendations of any guideline do not directly relate to the statistical significance of the literature findings. The panel is committed to providing indications to be used in clinical practice even when the published series are not large enough to reach statistical significance. On the other side, the panel may decide to be cautious in the presence of statistically significant data, considering other aspects within the Evidence-to-Decision process. For recommendation 2A (IPOMplus vs. IPOM), the panel concluded that the balance between desirable and undesirable effects justified the decision. However, the meta-analysis did not show statistically significant results (except for overall morbidity in the two RCTs, but not in the observational studies). On the contrary, the panel did not issue a recommendation in favor of the robotic repair (recommendation 2B—Robotic versus laparoscopic technique) despite the literature showing a statistically significant advantage for the robotic arm in more than one outcome because of equity and feasibility criteria (limited availability of the robotic technology). In the paper, we made every effort to clarify the reasons behind any decision.

In two instances, the panel could not provide a recommendation for the scarcity of the literature on the subject (recommendations 1E and 3).

This guideline's strengths include relying on an extensive systematic review of the literature and applying a rigorous GRADE method. It also has several limitations. The literature on the topic is continuously and rapidly evolving; our results are based on findings that need constant re-appraisal. The systematic review showed that high quality literature on the examined topics is scarce and the recommendations are mostly based on low certainty of evidence. This aspect must be considered in the decision-making process and has an impact on the practical application of the “conditional” recommendations. The guideline is focused only on minimally invasive techniques and did not consider broader issues (e.g., diagnostics, indication for surgery, pre-habilitation). In addition, several recently introduced minimally invasive techniques could not be included because, at present, they are only anecdotally reported in the literature.

Despite the described limitations and the limited evidence, the laparoscopic repair appears an alternative to open surgery within a tailored approach to each patient.

## Supplementary Information

Below is the link to the electronic supplementary material.

Supplementary file1 (DOC 45 KB)

## Data Availability

Data are available at this link https://www.iss.it/-/snlg-trattamento-laparoscopico-del-laparocele.
