# Supplementary material for: Laparoscopic treatment of ventral hernias: the Italian national guidelines
Source: Updates Surg. 2023 May 22;75(5):1305–36. doi: 10.1007/s13304-023-01534-3 (PMC10202362; doi:10.1007/s13304-023-01534-3)
Supplement: Supplementary file 1 — Supplementary file1 (DOC 45 KB) [file 13304_2023_1534_MOESM1_ESM.doc]

**Appendix 1.** Search strategies.

**Medline (via Pubmed)**

#39 #38 Filters: English, Italian, Humans, from 2000 – 2020 978

#38 #37 Filters: English, Italian, Humans

#37 #36 Filters: English, Italian

#36 #4 AND #7 AND #35

#35 #8 OR #9 OR #10 OR #11 OR #12 OR #13 OR #14 OR #15 OR #16 OR #17 OR

#18 OR #19 OR #20 OR #21 OR #22 OR #23 OR #24 OR #25 OR #26 OR #27 OR #28

OR #29 OR #30 OR #31 OR #32 OR #33 OR #34

#34 "Comparative Study" [Publication Type] #33 "Pragmatic Clinical Trials as Topic"[Mesh] #32 "Pragmatic Clinical Trial" [Publication Type]

#31 "Randomized Controlled Trials as Topic"[Mesh] #30 "Randomized Controlled Trial" [Publication Type]

#29 "Controlled Clinical Trials as Topic"[Mesh]

#28 "Controlled Clinical Trial" [Publication Type] #27 "Clinical Trials as Topic"[Mesh]

#26 "Clinical Trial" [Publication Type] #25 trial[Title/Abstract]

#24 random*[Title/Abstract]

#23 "Single-Blind Method"[MeSH Terms] #22 "Double-Blind Method"[MeSH Terms] #21 "Random Allocation"[MeSH Terms]

#20 "Randomized Controlled Trials as Topic"[MeSH Terms] #19 "Clinical Trials as Topic"[Mesh]

#18 "overview"[Title/Abstract]

#17 "review"[Title/Abstract]

#16 "metaanalysis"[Title/Abstract]

#15 "meta analysis"[Title/Abstract]

#14 "meta-analysis"[Title/Abstract]

#13 "Systematic Review"" [Publication Type]" #12 "Systematic Reviews as Topic"[Mesh]

#11 "Review"" [Publication Type]"

#10 "Review Literature as Topic"[Mesh] #9 "Meta-Analysis"" [Publication Type]" #8 "meta-analysis as topic"[MeSH Terms] #7 #5 OR #6

#6 laparosc*[Title/Abstract]

#5 "Laparoscopy"[MeSH Terms]

#4 #1 OR #2 OR #3

#3 (hernia* AND (ventral OR incisional OR epigastric OR umbilical OR parastoma* OR para-stoma* OR Spiegel* OR Spigel*))

#2 "Incisional Hernia"[Mesh Terms] #1 "hernia, ventral"[MeSH Terms]

**Embase (via Embase)**

#32 #7 AND #10 AND #29 AND ([english]/lim OR [italian]/lim) AND [2000-2020]/py AND [humans]/lim

#31#7 AND #10 AND #29 AND ([english]/lim OR [italian]/lim) AND [2000-2020]/py

#30 #7 AND #10 AND #29

#29 #11 OR #12 OR #13 OR #14 OR #15 OR #16 OR #17 OR #18 OR #19 OR #20 OR #21 OR #22 OR #23 OR #24 OR #25 OR #26 OR #27 OR #28

#28 trial:ab,ti

#27 random*:ab,ti

#26 ’randomization’/exp

#25 ’single blind procedure’/exp

#24 ’double blind procedure’/exp

#23 ’comparative study’/exp

#22 ’pragmatic trial’/exp

#21 ’randomized controlled trial’/exp

#20 ’controlled clinical trial’/exp

#19 ’clinical trial topic’/exp

#18 ’overview’:ab,ti

#17 ’review’:ab,ti

#16 ’metaanalysis’:ab,ti

#15 ’meta analysis’:ab,ti

#14 ’meta-analysis’:ab,ti

#13 ’systematic review’/exp

#12 ’review’/exp

#11 ’meta analysis’/exp

#10 #8 OR #9

#9 laparosc*

#8 ’laparoscopy’/exp

#7 #1 OR #2 OR #3 OR #4 OR #5 OR #6

#6 hernia* AND (ventral OR incisional OR epigastric OR umbilical OR parastoma* OR ’para stoma*’ OR spiegel* OR spigel*)

#5 ’spigelian hernia’/exp

#4 ’parastomal hernia’/exp

#3 ’epigastric hernia’/exp

#2 ’umbilical hernia’/exp

#1 ’incisional hernia’/exp

**Cochrane Library**

#1 MeSH descriptor: [Hernia, Ventral] explode all trees #2 MeSH descriptor: [Incisional Hernia] explode all trees

#3 ((hernia* AND (ventral OR incisional OR epigastric OR umbilical OR parastoma* OR para-stoma* OR Spiegel* OR Spigel*))):ti,ab,kw

#4 #1 OR #2 OR #3 1363

#5 MeSH descriptor: [Laparoscopy] explode all trees #6 laparosc*

#7 #5 OR #6

#8 #4 AND #7 with Cochrane Library publication date Between Jan 2000 and Jun 2020

**Web of Science (via Web of Science)**

| # 12 | #10 AND #2 AND #1 1,272  Refined by: LANGUAGES: ( ENGLISH OR ITALIAN ) Indexes=SCI-EXPANDED, CPCI-S Łimespan=2000-2020 |
| --- | --- |
| # 11 | #10 AND #2 AND #1  Indexes=SCI-EXPANDED, CPCI-S Łimespan=2000-2020 |
| # 10 | #9 OR #8 OR #7 OR #6 OR #5 OR #4 OR #3  Indexes=SCI-EXPANDED, SSCI, A&HCI, CPCI-S, CPCI-SSH, ESCI Łimespan=All years |
| # 9 | TOPIC: (trial)  Indexes=SCI-EXPANDED, SSCI, A&HCI, CPCI-S, CPCI-SSH, ESCI Łimespan=All years |
| # 8 | TOPIC: (random*)  Indexes=SCI-EXPANDED, SSCI, A&HCI, CPCI-S, CPCI-SSH, ESCI Łimespan=All years |
| # 7 | TOPIC: (overview)  Indexes=SCI-EXPANDED, SSCI, A&HCI, CPCI-S, CPCI-SSH, ESCI Łimespan=All years |
| # 6 | TOPIC: (review)  Indexes=SCI-EXPANDED, SSCI, A&HCI, CPCI-S, CPCI-SSH, ESCI Łimespan=All years |
| # 5 | TOPIC: (metaanalysis)  Indexes=SCI-EXPANDED, SSCI, A&HCI, CPCI-S, CPCI-SSH, ESCI Łimespan=All years |
| # 4 | TOPIC: (meta analysis)  Indexes=SCI-EXPANDED, SSCI, A&HCI, CPCI-S, CPCI-SSH, ESCI Łimespan=All years |
| # 3 | TOPIC: (meta-analysis)  Indexes=SCI-EXPANDED, SSCI, A&HCI, CPCI-S, CPCI-SSH, ESCI Łimespan=All years |
| # 2 | TOPIC: (laparosc*)  Indexes=SCI-EXPANDED, SSCI, A&HCI, CPCI-S, CPCI-SSH, ESCI Łimespan=All years |
| # 1 | TOPIC: ((hernia* AND (ventral OR incisional OR epigastric OR umbilical OR parastoma* OR para-stoma* OR Spiegel* OR Spigel*) ))  Indexes=SCI-EXPANDED, SSCI, A&HCI, CPCI-S, CPCI-SSH, ESCI Łimespan=All years |
